# Loss of central mineralocorticoid or glucocorticoid receptors impacts auditory nerve processing in the cochlea

**DOI:** 10.1016/j.isci.2022.103981

**Published:** 2022-02-26

**Authors:** Philine Marchetta, Philipp Eckert, Robert Lukowski, Peter Ruth, Wibke Singer, Lukas Rüttiger, Marlies Knipper

**Affiliations:** 1 University of Tübingen, Department of Otolaryngology, Head and Neck Surgery, Tübingen Hearing Research Centre, Molecular Physiology of Hearing, Elfriede-Aulhorn-Straße 5, 72076 Tübingen, Germany; 2 University of Tübingen, Institute of Pharmacy, Pharmacology, Toxicology and Clinical Pharmacy, 72076 Tübingen, Germany

**Keywords:** Molecular neuroscience, Cellular neuroscience, Sensory neuroscience

## Abstract

The key auditory signature that may associate peripheral hearing with central auditory cognitive defects remains elusive. Suggesting the involvement of stress receptors, we here deleted the mineralocorticoid and glucocorticoid receptors (**MR** and **GR**) using a CaMKIIα-based tamoxifen-inducible Cre^ERT2^/*loxP* approach to generate mice with single or double deletion of central but not cochlear MR and GR. Hearing thresholds of MRGR^CaMKIIαCreERT2^ conditional knockouts (**cKO**) were unchanged, whereas auditory nerve fiber (**ANF**) responses were larger and faster and auditory steady state responses were improved. Subsequent analysis of single MR or GR cKO revealed discrete roles for both, central MR and GR on cochlear functions. Limbic MR deletion reduced inner hair cell (**IHC**) ribbon numbers and ANF responses. In contrast, GR deletion shortened the latency and improved the synchronization to amplitude-modulated tones without affecting IHC ribbon numbers. These findings imply that stress hormone-dependent functions of central MR/GR contribute to “precognitive” sound processing in the cochlea.

## Highlights

• Top-down MR/GR signaling differentially contributes to cochlear sound processing
• Limbic MR stimulates auditory nerve fiber discharge rates
• Central GR deteriorates auditory nerve fiber synchrony

## Graphical abstract

**Figure:**
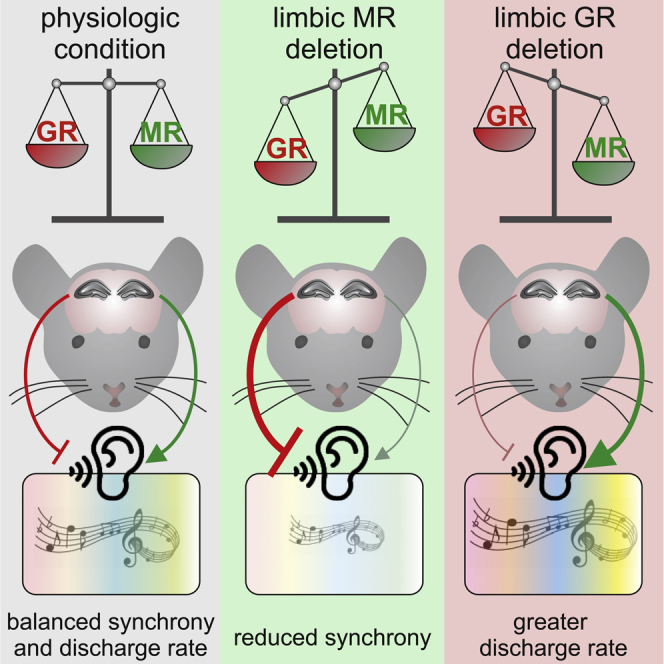

## Introduction

Hearing loss with age has recently been suggested to be an important modifying factor increasing the risk of dementia (Livingston et al., 2017; Montero-Odasso et al., 2020). The link between both pathologies is controversial. Until now, peripheral hearing and deficits in central cognitive processes, including cognitive decline and dementia, were predicted to be linked through limbic frontal brain dysfunction (Mudar and Husain, 2016), independent of cochlear functionality (Cope et al., 2015) and possibly disproportionate to peripheral hearing loss (Johnson et al., 2021). Among others, central hearing deficits include deficits in attention or executive function (Rutherford et al., 2018) that are also altered through chronic stress (Perez-Valenzuela et al., 2019; Panza et al., 2019; Canlon et al., 2013). Blockade of stress-hormone binding mineralocorticoid receptors (**MR**), for instance, impairs both memory tasks and selective attention (Wingenfeld and Otte, 2019; de Kloet et al., 2000, 2019). On the other hand, through neuronal atrophy and synaptic dysfunction, chronic stress can contribute to the degradation of synaptic plasticity and thereby influence cognitive functions (for review see Vyas et al., 2016). In the auditory system, numerous studies that analyzed stress-related hearing dysfunction in response to age, acoustic trauma (**AT**), or posttraumatic stress observed associated changes in cognitive functions (Jafari et al., 2019; Basner et al., 2014; Mazurek et al., 2019; Nadhimi and Llano, 2021; Johnson et al., 2021; Wang and Puel, 2020; Canlon et al., 2013; Meltser and Canlon, 2011). How stress-receptor activation links hearing and cognition is, however, currently elusive.

Glucocorticoid receptors (**GR**) and MR in the limbic system, including the prefrontal cortex and hippocampus, are suggested to mediate the top-down and bottom-up control of stress coping with environmental challenges through hypothalamic and extrahypothalamic prefrontal and hippocampal regions (de Kloet et al., 2000, 2019). Previous studies implied that pharmacological or acoustic trauma-induced stress affects central auditory processing through sensorineural cochlear responses (Singer et al., 2013, 2018). To examine whether the brain’s ability to recognize and interpret sound depends on stress hormone receptors, we tested the effect of induced genetic disruption of central MR and GR on cochlear function. Employing the tissue-specific tamoxifen (**TMX**)-inducible Cre^ERT2^/*loxP* system allowed for single and combined deletion of MR and GR in glutamatergic forebrain neurons under the promoter of CaMKIIα in adult mice (conditional MRGR^CaMKIIαCreERT2^ knockout; **MRGR cKO**). This leads to deletion of MR and GR, mainly in the forebrain, with preference for the limbic system, as high levels of CaMKIIα can be found in the hippocampus, cortex, and amygdala; lower levels of CaMKIIα are expressed in striatum, thalamus, and hypothalamus, while CaMKIIα is not present in the cerebellum or outside of the brain (Erdmann et al., 2007). Given that GR are expressed in virtually all cell types in the rodent brain, and MR are expressed primarily in neurons of limbic regions such as the hippocampus, lateral septum, and amygdala (de Kloet et al., 2005; McEwen et al., 2016; Chao et al., 1989; Reul and de Kloet, 1985), while both MR and GR are expressed in cochlear hair cells, supporting cells, and spiral-ganglion neurons (Kil and Kalinec, 2013; Yao and Rarey, 1996; Erichsen et al., 2001; ten Cate et al., 1992, 1993; Zuo et al., 1995), this targeting strategy allowed us to test the specific influences of central stress receptors on peripheral cochlear function.

GR and MR differ in their glucocorticoid (**GC**) binding affinity (de Kloet et al., 2005). MR are highly affine for the endogenous GC cortisol and corticosterone (**CORT**; the latter is the predominant GC in mice), with an approximate Kd (dissociation constant) of 0.5 nM (Reul et al., 1990), which makes MR responsive to acute and mild stress events that are relevant for proper neural responses of learning, memory, and selective attention to novel situations (Joels and de Kloet, 2017; Wirz et al., 2017; Plieger et al., 2018). Compared to MR, GR have only a 10th of the affinity (Kd ≈ 5 nM) for GC. GR play an important role in memory consolidation and long-time adaptation to stressful situations (de Kloet et al., 2005); in addition, because of their widespread expression, are most reactive during chronic stress responses (Sapolsky, 2015; de Kloet et al., 1999, 2005). Thus, assessing specific single MR^CaMKIIαCreERT2^ knockout (**MR cKO**) or GR^CaMKIIαCreERT2^ knockout (**GR cKO**) mutant mice should give us insights into the common or distinct role of central MR and GR in peripheral cochlear function.

In the present study we observed that the double-deletion of MR and GR strikingly improved cochlear sensitivity, as measured by auditory brainstem response (**ABR**) wave amplitude, compound action-potential (**CAP**) threshold and latency, and neural temporal sound processing underlying auditory steady state responses (**ASSR**). This occurred independently of changes in the mechanics of cochlear outer hair cells (**OHC**) as measured via distortion-product otoacoustic emissions (**DPOAE**), reflecting a negative top-down action of limbic forebrain stress receptors on high-fidelity signal coding and fast auditory processing. The phenotype of MRGR cKO mice combined the unfavorable effects of MR deletion on inner hair cell (**IHC**) ribbon numbers that determine the discharge rate of auditory nerve fibers (**ANF**) on the one hand, with positive effects of GR deletion on CAP latencies and ASSR, influencing temporal auditory coding on the other. Apparently, limbic forebrain MR and GR activities can directly improve or weaken, respectively, the temporal power of sound processing at the level of the cochlea. We suggest that central, i.e., forebrain limbic MR and GR activity, can influence the cochlear sound processing through its top-down influence.

## Results

### Deletion of MR and GR in CaMKIIα-expressing forebrain regions but not in the cochlea

The Cre^ERT2^-dependent deletion of the stress receptors MR and GR was performed under control of the CaMKIIα promoter (Erdmann et al., 2007), which is expressed in the whole forebrain, but with highest density in the hippocampus (Dragatsis and Zeitlin, 2000; Wang et al., 2013). To validate the TMX-induced Cre^ERT2^-directed recombination, the Rosa^tdTomato^ Cre-reporter strain (Madisen et al., 2010) was crossed with CaMKIIα-Cre^ERT2^ mice. Double-transgenic CaMKIIα-Rosa^tdTomato^ mice were examined for Cre^ERT2^-mediated expression of endogenous red fluorescence in the hippocampus and the cochlea. Although staining was absent in Cre^ERT2^ negative mice (Figure S1A, left panel), strong endogenous red tdTomato fluorescence was found in the hippocampus of Cre^ERT2^ positive CaMKII-Rosa^tdTomato^ mice (Figure S1A, right panel). In neither Cre positive nor Cre negative Rosa^tdTomato^ mice was red fluorescence detectable in cochlear whole-mounts (Figure S1B), indicating that CaMKIIα-Cre^ERT2^ was not activated by TMX injection in the cochlea.

**Figure 1:**
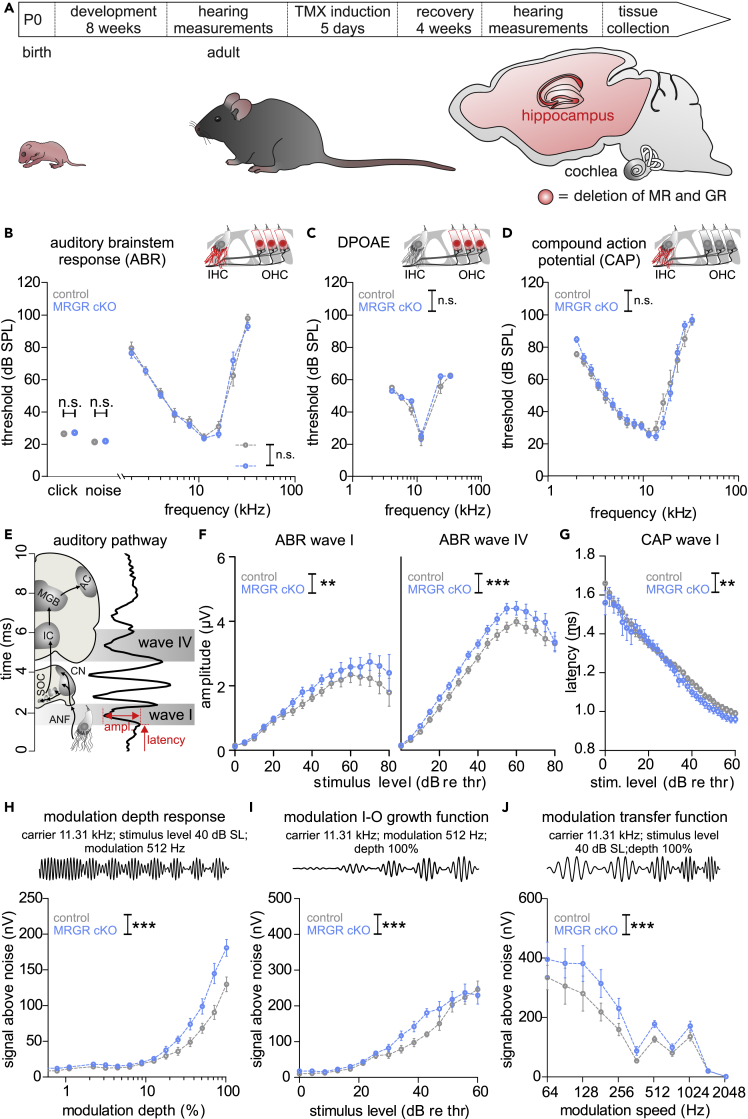
Improved suprathreshold hearing function in MRGR cKO (A) Workflow of the experiment. 8 weeks after birth, adult, developed mice were checked for hearing function and injected with tamoxifen (TMX). After 4 weeks of recovery, the hearing was measured again and mice were sacrificed to collect brain and cochlea tissue. The TMX induction led to a tissue-specific deletion of MR and GR under the promoter of CaMKIIα, which is expressed in the forebrain, but not the cochlea. (B) No difference between thresholds in MRGR cKO and control mice with click- (unpaired Student’s *t* test, t(50) = 0.620, p = 0.538, WT: n = 12/24, KO: n = 14/28 mice/ears), noise-burst evoked auditory brainstem responses (**ABR**; t(50) = 0.451, p = 0.653, WT: n = 12/24, KO: n = 14/28 mice/ears), as well as pure tone ABR (two-way ANOVA, F(1,8) = 0.191, p = 0.663, WT: n = 10, KO: n = 11 mice/ears). (C) No difference between thresholds in MRGR cKO and control mice in distortion-product otoacoustic emission (**DPOAE**) thresholds (two-way ANOVA, F(1,5) = 1.772, p = 0.186, WT: n = 5/10, KO: n = 8/16 mice/ears). (D) No difference between CAP thresholds (two-way ANOVA, F(1,16) = 0.368, p = 0.5443, WT: n = 5/9, KO: n = 7/13 mice/ears) between MRGR cKO and control mice. (E) Schematic drawing of the auditory pathway and correlated ABR waves. (F) Left panel: Increased ABR wave I amplitude in MRGR cKO (two-way ANOVA, F(1,17) = 10.80, p = 0.0011, WT: n = 12/24, KO: n = 15/30 mice/ears) and (right panel) increased ABR wave IV amplitude (two-way ANOVA, F(1,17) = 31.62, p < 0.0001, WT: n = 12/24, KO: n = 14/28 mice/ears) compared to controls. (G) Shortened CAP latency of wave I in MRGR cKO mice (two-way ANOVA, F(1,620) = 70.94, p = 0.0022, WT: n = 6/11, KO: n = 7/13 mice/ears) as compared to controls. (H) Larger modulation depth function in MRGR cKO mice (two-way ANOVA, F(1,572) = 11.38, p = 0.0008, WT: n = 19, KO: n = 18 mice/ears). (I) Increased growth function in MRGR cKO mice (two-way ANOVA, F(1,688) = 5.210, p = 0.023, WT: n = 18, KO: n = 20 mice/ears) compared to controls. (J) Increased modulation transfer function in MRGR cKO mice (two-way ANOVA, F(1,473) = 14.37, p = 0.0002, n = 19 mice/ears) compared to WT mice. Mean ± SEM. ∗ = p < 0.05, ∗∗ = p < 0.01, ∗∗∗ = p < 0.001, n.s. = not significant (p > 0.05). AN = auditory nerve, CN = cochlear nucleus, SOC = superior olivary complex, IC = inferior colliculus, MGB = medial geniculate body, AC = auditory cortex.

Next, we induced the deletion of MR and GR by TMX injection (5 days, twice daily 1 mg i.p.) in adult pre-mutants bearing floxed MRGR alleles (Figure 1A). In hippocampal pyramidal neurons of the CA1 region, anti-MR staining (Figure S1C, green), as well as anti-GR staining (Figure S1D, green), was only seen in control mice (left panel), but not in MRGR cKO mice (right panel) receiving TMX.

In contrast, MR expression at the level of OHC was found in both control and MRGR cKO mice (Figure S1E, green), whereas GR labeling was seen in the cochlea at the level of IHC and OHC. This labeling was again not different between control and CaMKIIα-Cre^ERT2^-mediated conditional MRGR mutants (Figure S1F, green).

We conclude that our TMX-induction protocol promotes the activation of CaMKIIα-Cre^ERT2^, which leads to an efficient deletion of MR and GR specifically in limbic brain regions (hippocampus) (Erdmann et al., 2007), but that TMX injection did not delete these receptors in the auditory periphery, as schematically depicted in Figure 1A.

### Deletion of MR and GR in MRGR cKO mice exposed a negative impact of stress receptors on auditory temporal processing, independently of OHC

We demonstrated that the hearing of Cre positive and Cre negative animals bearing floxed MRGR alleles was not different in the absence of TMX (data not shown). In addition, after TMX injection, ABR thresholds of control and MRGR cKO mice were not different, as shown for click-evoked and noise-evoked responses and for pure-tone stimuli between 2 and 32 kHz (Figure 1B). When DPOAE thresholds were analyzed, providing information about electromechanical properties of OHC function, no difference between the genotypes was observed (Figure 1C). Likewise, the population response thresholds to sound from ANF were not different between the two genotypes (Figure 1D). In contrast, suprathreshold click-evoked ABR that are calculated as peak-to-peak amplitude growth functions of ABR wave I (originating from the auditory nerve) and ABR wave IV (originating from the inferior colliculus) (Melcher et al., 1996), were significantly increased in MRGR cKO mice (Figures 1E and 1F, left and right panel). Thus the stimulus-level-dependent spreading of sound-response amplitudes was already elevated in the auditory periphery, where MR and GR levels were unaltered in MRGR cKO (Figure S1B).

The effects of simultaneous MR deletion and GR deletion on auditory-nerve processing were confirmed by the shorter CAP response latencies in MRGR cKO mice in comparison to control mice, especially at stimulus levels >30 dB above the threshold (Figure 1G). Moreover, stronger ASSR, used as a measure for temporal sound-coding capacity, as described in (Möhrle et al., 2016), was observed in MRGR cKO mice (Figures 1H–1J). ASSR are periodic electrical brain oscillations induced by acoustic stimuli and sinusoidally modulated in amplitude and frequency (Picton et al., 2003). They reflect the synchronous discharge of auditory neurons phase-locked to the modulation frequency of the stimulus (Brenner et al., 2009; Dolphin and Mountain, 1992; Kuwada et al., 2002; Parthasarathy and Bartlett, 2012). We used a carrier frequency of 11.32 kHz that was modulated by a second, slower frequency, ranging between 64 and 2048 Hz, with a modulation depth between 1 and 100% and a stimulus level ranging between 0 and 60 dB above ABR threshold. To this stimulus, MRGR cKO mice showed a significantly enhanced response compared with control mice with regard to modulation depth (Figure 1H), growth rate with stimulus level (Figure 1I), and modulation speed (Figure 1J).

Together, these findings imply that the acute, combined deletion of MR and GR under the CaMKIIα promoter, while not influencing the electromechanical properties of OHC, leads to faster and more sensitive sensorineural cochlear processing, resulting in elevated brain responses to amplitude-modulated tones. This in turn suggests that the combined MR/GR activation during, e.g., elevated (chronic) stress might inhibit auditory neuronal synchronization and temporal auditory processing.

### Differential influences of MR or GR on discharge rate and synchrony of auditory nerve responses contribute to the overall phenotype of MRGR cKO on auditory processing

**Figure 2:**
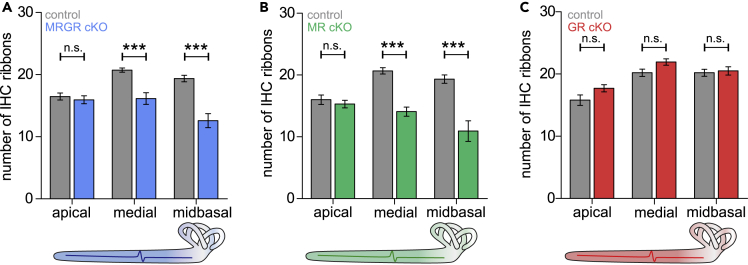
MRGR cKO mice and MR cKO mice, but not GR cKO mice, show reduced numbers of IHC ribbon synapses (A) Reduced numbers of IHC ribbons in medial and midbasal turns of MRGR cKO mice (two-way ANOVA, F(1,2) = 41.10, p < 0.0001, WT vs. KO apical: p > 0.05, medial: p < 0.001, midbasal: p < 0.001, WT: n = 19/9, KO: n = 18/9 IHC/mice). (B) Reduced numbers of IHC ribbons in medial and midbasal turns of MR cKO mice (two-way ANOVA, F(1,2) = 44.25, p < 0.0001, WT vs. KO apical: p > 0.05, medial: p < 0.001, midbasal: p < 0.001, WT: n = 12/5, KO: 14/5 IHC/mice). (C) No difference in IHC ribbons numbers between GR cKO and control mice (two-way ANOVA, F(1,2) = 6.603, p = 0.0125, WT vs. KO apical: p > 0.05, medial: p > 0.05, midbasal: p > 0.05, WT: n = 10/5, KO: n = 14/6 IHCs/mice). Mean ± SEM. ∗∗∗ = p < 0.001, n.s. = not significant (p > 0.05).

As a possible rationale for the observed altered auditory responses in the MRGR cKO mice, we considered differences in the number of IHC ribbons, which influence auditory processing through defining an ANF’s discharge rate (Kujawa and Liberman, 2009; Buran et al., 2010). To examine the ribbons, we used antibodies directed against the RIBEYE protein CtBP2 (Khimich et al., 2005). In MRGR cKO mice, IHC ribbon numbers were significantly reduced in high-frequency coding medial and midbasal cochlear turns (Figure 2A). It is assumed that IHC ribbons can be subdivided into modiolar-sided vs. pillar-sided ribbon synapses, which contact postsynapses that differ functionally in spontaneous firing rates (**SR**) and thresholds for sound coding. The large modiolar ribbon synapses are known to have contact with small postsynapses of low-SR, high-threshold ANF, which are recruited at higher SPL and show little or no saturation. The small pillar ribbon synapses have contact with large postsynapses of high-SR, low-threshold ANF, which are activated in response to lower SPL and rapidly saturate (Liberman, 1982; Liberman and Oliver, 1984; Merchan-Perez and Liberman, 1996; Winter et al., 1990). In C57BL/6 mice the ratio between modiolar-sided and pillar-sided IHC ribbons is around 50:50 (Reijntjes et al., 2020). The same proportion was confirmed in MRGR control mice when analyzing the modiolar/pillar gradient of IHC ribbons in the midbasal turn (Figure S2A). However, MRGR cKO had less ribbons located on the pillar side of the IHC in comparison to ribbons counted on the modiolar side (Figure S2B; left panel). This was contrary to assumptions, as typically, a larger ABR wave I is expected to correlate with a larger, and not a reduced, number of IHC ribbons (Buran et al., 2010; Kujawa and Liberman, 2009). These inconsistencies might have their rational in an overlap of contrasting MR-influences or GR-influences on sensorineural responses (see below).

We hypothesized that a combined MRGR cKO may uncover possible differential effects of the individual MR or GR functions on auditory processing. Indeed, examining TMX-inducible single MR cKO and GR cKO mice revealed that IHC ribbons in MR cKO mice were like in MRGR cKO mice numerically reduced (Figure 2B) and exhibited in high frequency cochlear regions a smaller number of IHC ribbons on the pillar vs. the modiolar side (Figure S2B; middle panel). In contrast, in GR cKO mice, no changes of IHC ribbon numbers (Figure 2C) between controls and GR cKO mice were seen, and the percentage of IHC ribbons numbers on pillar vs. modiolar sides in midbasal turns were not different in WT and GR cKO mice (Figure S2B; right panel). This may suggest that the observed reduction of IHC ribbon numbers, seen in MRGR cKO mice, is rather linked to the deletion of central MR.

When putative effects of the distinct limbic MR and GR ablation on ABR and DPOAE thresholds were analyzed, the ABR hearing thresholds in response to click-tone, noise-tone, or pure-tone stimuli (Figures S3A and S3C), as well as DPOAE thresholds (Figures S3B and S3D), were not found to be different in MR cKO nor GR cKO compared to their respective controls. This supports the notion that neither the limbic MR nor GR affects basal hearing thresholds or electromechanical OHC response properties.

**Figure 3:**
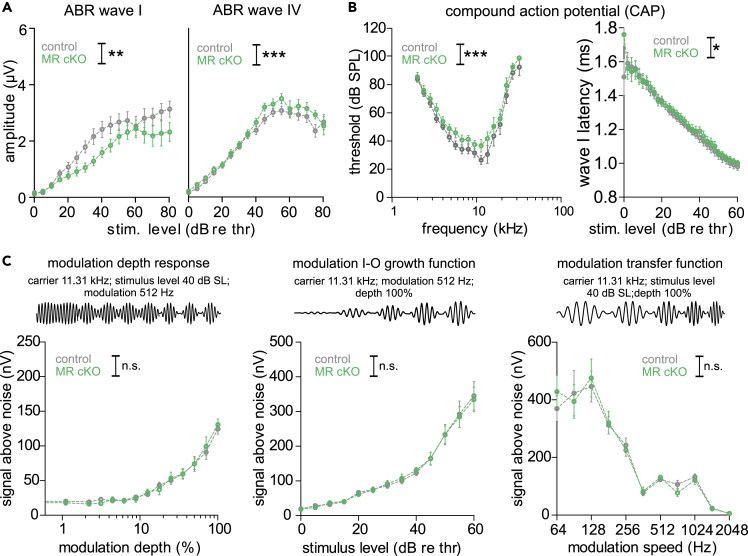
Reduced hearing function in MR cKO (A) Reduced ABR wave I amplitude (left panel, two-way ANOVA, F(1,18) = 19.40, p < 0.0001, WT: n = 8/16, KO: n = 8/16 mice/ears), but increased ABR wave IV amplitude MR cKO compared with WT mice (right panel, two-way ANOVA, F(1,17) = 6.991, p = 0.0085, WT: n = 8/16, KO: n = 8/16 mice/ears). (B) Left panel: Increased CAP pure tone-evoked threshold in MR cKO (two-way ANOVA, F(1,16) = 14.92, p = 0.0001, n = 7/14 mice/ears). Right panel: Extended CAP wave I latency in MR cKO (two-way ANOVA, F(1,733) = 5.57, p = 0.0186, WT: n = 7/14, KO: n = 6/11 mice/ears). (C) No difference in modulation-depth function (two-way ANOVA, F(1,701) = 0.14, p = 0.999, WT: n = 24, KO: n = 21 mice/ears), stimulus growth function (two-way ANOVA, F(1,955) = 0.01, p = 1.000, WT: n = 24, KO: n = 22 mice/ears), and modulation transfer function (two-way ANOVA, F(1,583) = 0.02, p = 0.8905, WT: n = 24, KO: n = 22 mice/ears) between MR cKO and control mice. Mean ± SEM. ∗ = p < 0.05, ∗∗ = p < 0.01, ∗∗∗ = p < 0.001, n.s. = not significant (p > 0.05).

**Figure 4:**
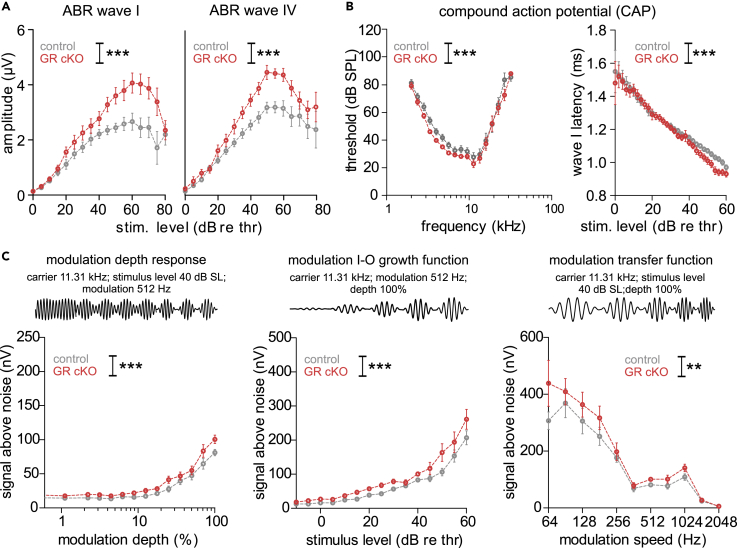
Improved hearing function in GR cKO (A) Increased ABR wave I (left panel, two-way ANOVA, F(1,17) = 45.83, p < 0.0001, WT: n = 7/14, KO: n = 8/16 mice/ears) and ABR wave IV amplitudes (right panel, two-way ANOVA, F(1,17) = 57.51, p < 0.0001, WT: n = 7/14, KO: n = 8/16 mice/ears) in GR cKO compared with control mice. (B) Left panel: Improved CAP threshold in GR cKO mice (two-way ANOVA, F(1,16) = 14.60, p = 0.0001, WT: n = 8/15, KO: n = 9/18 mice/ears). Right panel: Shortened CAP wave I latency in GR cKO mice (two-way ANOVA, F(11,043) = 14.04, p = 0.0002, WT: n = 10/20, KO: n = 10/19 mice/ears). (C) Larger signal-to-noise ratio (two-way ANOVA, F(1,780) = 15.56, p < 0.0001, WT: n = 26, KO: n = 24 mice/ears), growth function (two-way ANOVA, F(11,025) = 34.58, p < 0.0001, WT: n = 30, KO: n = 28 mice/ears), and modulation transfer function (two-way ANOVA, F(1,636) = 9.99, p = 0.0017, WT: n = 26, KO: n = 24 mice/ears) in GR cKO mice compared with control mice. Mean ± SEM. ∗∗ = p < 0.01, ∗∗∗ = p < 0.001.

The reduction of IHC ribbons in MR cKO mice was linked to a reduction of the click-evoked ABR wave I amplitude when compared to controls (Figure 3A, left panel). This was, however, centrally compensated, as evident from the normal-sized or even larger ABR wave IV (right panel). The smaller ABR wave I response in MR cKO mice was functionally reflected in slightly, but significantly, higher CAP thresholds compared to control mice (Figure 3B, left panel), and in slightly prolonged CAP latencies (right panel). When ASSR were analyzed in MR cKO mice, we observed no difference to control mice in the modulation-depth function at 40 dB relative to threshold (Figure 3C, left panel), in modulation growth functions (middle panel), or in the modulation transfer function (right panel). This together suggested that the influence of MR deletion on auditory processing is restricted to IHC ribbon numbers and ABR wave I amplitudes. In contrast, the deletion of limbic GR, while leaving IHC synapse ribbon numbers unaffected, led to higher click-evoked ABR wave I (Figure 4A, left panel) and wave IV amplitudes (right panel), as also observed in MRGR cKO mice (Figure 1F). Consistently, compared with control mice, GR cKO mice exhibited a significantly lower CAP threshold (Figure 4B, left panel), and shorter CAP wave I latency (right panel). The shorter CAP latency in GR cKO mice was coincident with stronger ASSR responses, evident in a larger signal than in controls when measuring responses to amplitude-modulated stimuli with variation in modulation depth (Figure 4C, left panel), modulated in stimulus level (Figure 4C, middle panel, or changes in modulation frequency (Figure 4C, right panel). Thus, GR deletion influences auditory processing through changes in spike timing and in the synchronization of neural auditory responses that were shown to be required for following amplitude-modulated stimuli (Johnson, 1980).

Overall, these findings in MRGR cKO mice suggested a negative influence of limbic MR deletion on auditory nerve responses, evidenced through reduced IHC ribbon numbers (Figure 2), reduced ABR wave amplitudes, elevated CAP thresholds, and prolonged CAP latencies in MR cKO mice (Figure 3). This negative effect was counterbalanced after central GR loss that – as shown in GR cKO mice – results in elevated ABR amplitudes, lower CAP thresholds, and shortened CAP latencies, as well as facilitated ASSR (Figure 4). Both phenotypes together, reduction of the peripheral auditory processing after MR deletion and stimulation after GR deletion, contribute to the complex phenotype of MRGR cKO mice (Figure 1).

### Blood corticosterone levels do not account for the differential effects on peripheral hearing in MR cKO, GR cKO, and MRGR cKO mice

**Figure 5:**
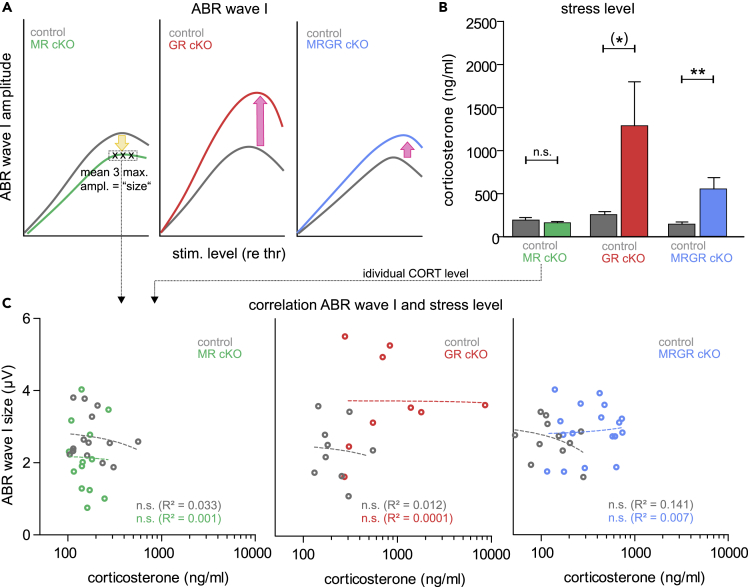
Corticosterone (CORT) levels do not correlate with wave I amplitude (A) Schematic drawing of reduced ABR wave I amplitude in MR cKO (left panel), strongly increased ABR wave I amplitude in GR cKO (middle panel), and increased ABR wave I amplitude in MRGR cKO mice (left panel). (B) Similar CORT levels in MR cKO and control mice (unpaired Student’s *t* test, t(28) = 90.9285, p = 0.3611, WT: n = 16, KO: n = 14 mice). Increased CORT levels in GR cKO mice, compared with control mice (unpaired Student’s *t* test, t(28) = 1.884, p = 0.070, WT: n = 14, KO: n = 16 mice). Increased CORT levels in MRGR cKO mice, compared with control mice (unpaired Student’s *t* test, t(24) = 2.905, p = 0.0078, WT: n = 12, KO: n = 14 mice). (C) Left panel: No correlation for MR cKO and control mice between ABR wave I amplitude and CORT level (linear regression; WT: R^2^ = 0.033, p = 0.5329, KO: R^2^ = 0.001, p = 0.9322, WT: n = 14, KO: n = 13 mice). Middle panel: No correlation for GR cKO and control mice between ABR wave I amplitude and CORT level (linear regression; WT: R^2^ = 0.012, p = 0.7802, KO: R^2^ = 0.0001, p = 0.9723, n = 9 mice). Right panel: No correlation for MRGR cKO and control mice between ABR wave I amplitude and CORT level (linear regression; WT: R^2^ = 0.141, p = 0.2540, KO: R^2^ = 0.007, p = 0.7574, WT: n = 11, KO: n = 17 mice). Mean ± SEM. (∗) = p < 0.1, ∗∗ = p < 0.01, n.s. = not significant (p > 0.05).

Previously, the spatiotemporal deletion of GR in limbic forebrain regions was shown to enhance blood CORT levels through an unbalanced autoregulation of hippocampal GR on the hypothalamus-pituitary-adrenal (**HPA**)-axis (Erdmann et al., 2007). To investigate to what extent TMX-induced deletion of MR/GR might also exhibit its influence through an unbalanced HPA-axis, we analyzed the CORT level from blood plasma in anesthetized MR cKO, GR cKO, MRGR cKO, and control mice during hearing measurements. In MR cKO mice that exhibited smaller ABR wave amplitudes (Figure 5A, left panel), the level of CORT was identical to that of controls (Figure 5B, left panel), and the ABR wave I amplitude size, which was the mean of the three maximal amplitude values of the individual ears' suprathreshold amplitude growth function, was not correlated with an individual animal’s stress-hormone levels (Figure 5C, left panel). In contrast, in GR cKO mice, characterized by an elevated ABR wave I (Figure 5A, middle panel), a tendency toward higher CORT levels was observed (Figure 5B, middle panel). Again, no direct correlation between ABR wave I amplitude size and an individual animal’s stress levels was found (Figure 5C, middle panel). In MRGR cKO both ABR wave I (Figure 5A, right panel) and the CORT level were elevated (Figure 5B, right panel). However, no correlation between the ABR wave I amplitude size and an individual animal’s stress hormone levels was found (Figure 5C, right panel). This may suggest that the GR-deletion-induced HPA-axis dysregulation is not the primary driver for the observed changes of MR/GR cKO on peripheral hearing.

**Figure 6:**
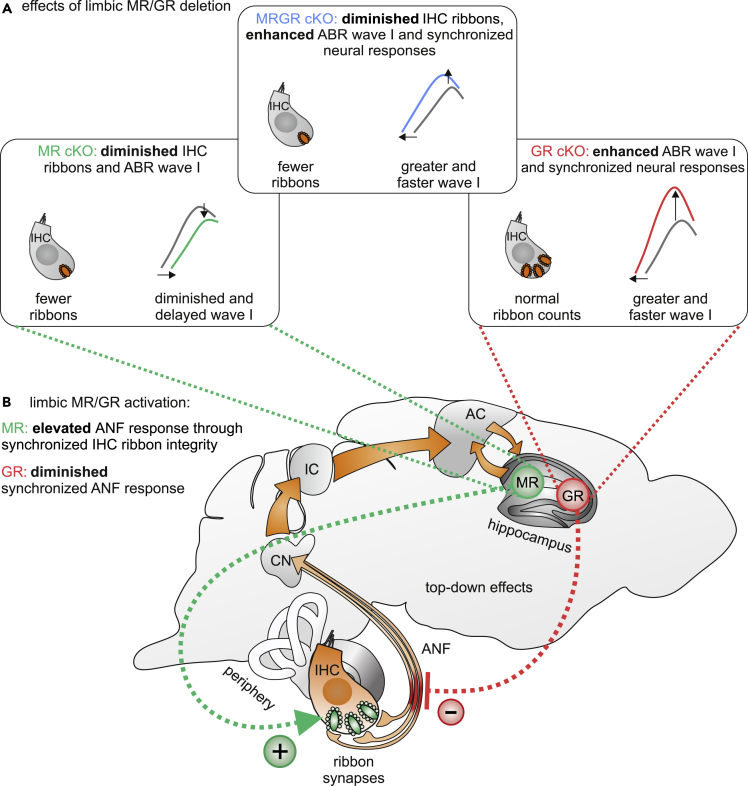
Hypothesized effect of limbic MR/GR activation on auditory processing in the cochlea (A) Compound limbic deletion of MR and GR leads to reduced IHC ribbon numbers and enhanced auditory neural responses to modulated tones, indicating improved synchrony of neural responses. This phenotype is a mixture of MR cKO mice with reduced IHC ribbon numbers, reduced ABR wave I, but without effects on synchronized neural auditory responses, and GR cKO mice with normal IHC ribbon numbers and increased ANF synchrony. (B) Hypothesized physiological effect of the activation of limbic MR, possibly contributing to discharge rate, stabilized presynaptic IHC elements, and thereby to an improved ASSR, whereas the activation of limbic GR leads to the inhibition of ANF synchrony, thereby diminishing temporal auditory coding through degradation of synchronized neural auditory responses

In conclusion, GC activation works in a binary fashion on IHC synapses and auditory-nerve synchrony: by activation of either the stimulating limbic MR (Figure 6, green plus) on the one hand and by activation of the inhibiting GR (Figure 6, red minus) on the other hand. Signaling through these receptors thus provides a key auditory signature that may associate peripheral hearing with central auditory cognitive hearing defects (Figure 6).

## Discussion

Physical and psychological stressors are manifested through the activation of the HPA-axis, and the production of GC exerts profound effects on neuronal networks and sensory gating. Acutely elevated and chronically elevated GC levels are assumed to influence sensory gating at the cortical or hippocampal level, independent of peripheral sensory function (Johnson et al., 2021; Basner et al., 2014). By investigating TMX-induced conditional single or combined deletion of MR/GR in forebrain regions, we report functional roles of limbic MR and GR as peripheral modulators of the IHC synapse and in ANF processing. This provides a concept for limbic MR and GR activities during acute or chronic stress, which should be reconsidered as modulators for subcortical processing and filtering elements during auditory perception. In this context, either limbic MR or GR function contribute to the precision of auditory processing, thereby possibly influencing speech comprehension and cognitive hearing function.

### Specificity of the TMX–induced limbic deletion of MR and GR

Homozygous global GR KO (Cole et al., 1995), as well as Cre-mediated early embryonic ablation of GR by a constitutively active Cre in GR^CaMKIIα^ KO mice (Erdmann et al., 2008), are lethal. As early as P8, animals with germline inactivation of MR, as shown in the homozygous global MR KO, suffer from hyperkalemia, hyponatremia, weight loss, and a strong increase in renin, angiotensin II, and aldosterone plasma concentrations. Consequently, a global lack of MR induces early postnatal death because of bodily dehydration (exsiccosis) as a consequence of massive renal sodium and water loss (Berger et al., 1998). To overcome the lethal phenotype of global GR KO and cell-specific (non-inducible) GR^CaMKIIα^ KO, and the severely diseased phenotype of young MR KO (Erdmann et al., 2007; Berger et al., 1998), we used TMX-inducible Cre driver lines (Berger et al., 2006; Erdmann et al., 2008) for the generation of adult MR cKO, GR cKO, and MRGR cKO mouse models. This enabled in parallel the extraction of specific roles of GR and MR in hearing in adulthood. As shown for MRGR cKO mice, both target genes were deleted in pyramidal cells of the forebrain (Figures S1C and S1D), but not in the cochlea (Figures S1E and S1F). The absence of CaMKIIα in the cochlea is in line with other studies (Meese et al., 2017). We observed high CORT levels in MRGR cKO mice, and partly also in the GR cKO, which corresponds well with an analogous GR^CaMKIIα^ KO mouse line generated by Erdmann and coworkers, whereas no increase was seen in MR cKO mice (Figure 5A) (Erdmann et al., 2007). This suggests that the TMX-inducible double deletion and single deletion of MR and GR mirrors previous mouse models with respect to HPA-axis dysfunction. However, GR cKO and MR cKO mice presented with distinct hearing phenotypes, which is in line with predictions that GR and MR serve nonredundant functions in neurons to control distinct transcriptional networks (Mifsud and Reul, 2016; Obradovic et al., 2004; de Kloet et al., 2000; McCann et al., 2021; Reul and de Kloet, 1985). As both nuclear receptors recognize the same specific DNA promoter sequences, called GC response elements (Rupprecht et al., 1993; Mifsud and Reul, 2016), the differential effects of MR and GR may stem from the different MR/GR expression profiles. GR is expressed in virtually all cell types in the rodent brain, whereas MR is expressed primarily in neurons of limbic regions such as the hippocampus, lateral septum, and amygdala (de Kloet et al., 2000; Chao et al., 1989; Reul and de Kloet, 1985). Besides their individual expression profile across the brain, differential MR and GR effects derive from different affinities of the individual receptors, with MR having a 10-fold higher affinity for CORT than GR, being occupied by ligands even under baseline, low-stress conditions (Arriza et al., 1988; Gomez-Sanchez and Gomez-Sanchez, 2014). GR, in contrast, are activated when the animal is stressed or during circadian periods when circulating CORT levels are naturally elevated (Mifsud and Reul, 2016; Joels et al., 2008; de Kloet et al., 1993).

Because MR has the same binding affinity for aldosterone, cortisol, and CORT (Gomez-Sanchez and Gomez-Sanchez, 2014), changes in presumptive aldosterone effects on hearing might contribute substantially to the MR cKO phenotype. Until now, only long-term effects of aldosterone on hearing have been described, acting through influences on the endocochlear potential (Bazard et al., 2020). The inactivation of cortisol and CORT by 11beta-hydroxysteroid dehydrogenase type 2 (**11beta-HSD2**) is, moreover, required to allow aldosterone to activate MR within aldosterone target cells (Gomez-Sanchez and Gomez-Sanchez, 2014). An immunoreactivity of 11beta-HSD2 has, however, not been detected in any inner ear tissues (Terakado et al., 2011).

Finally, regarding the prominent circadian activity exhibited by the HPA-axis, functional hearing experiments were conducted during the same time period of the day. We were thereby able to avoid dynamic changes in the intrinsic properties of these MR-positive and/or GR-positive neural populations, which may alter the function of neural circuits affecting central or peripheral auditory pathways.

Together, the distinct central and cochlear MR/GR expression profiles, MR/GR-dependent changes in blood CORT levels, as well as the apparently contrasting auditory functions of limbic MR and GR, mirror, at least partly, the respective phenotypes of global and conditional MR/GR KO (Cole et al., 1995; Erdmann et al., 2008). Combined, these findings substantiate the specific MR and GR deletion in limbic forebrain regions of these mouse models.

### Differential impact of limbic MR and GR activation on auditory-nerve processing is independent of OHC

Activation of both MR and GR in frontal brain regions and the hippocampus has a direct and differential impact on peripheral auditory processing. This can be concluded from the observation that the induced, combined limbic deletion of MR and GR in adult animals significantly enhanced amplitudes and reduced delays in auditory-nerve responses, independently of OHC function, as no difference was found in the DPOAE measurements of control and MRGR cKO mice (Figure 1). The amplitude of the suprathreshold evoked ABR wave I (Kujawa and Liberman, 2009), the peripheral neural response that is affected in MR and GR cKO mice, is determined through discharge rate and synchrony changes of the auditory nerve (Johnson and Kiang, 1976; Kujawa and Liberman, 2009). ABR wave I spreads centrally to generate ABR wave IV at the level of the inferior colliculus (Melcher et al., 1996). At the cochlear level the effect of MR/GR deletion was quantified through the altered threshold and latency of acoustically-evoked CAP responses reflecting summed single-fiber action potentials of ANF (Earl and Chertoff, 2010; Rüttiger et al., 2017). These CAP measurements allow, moreover, a first estimate of possible distinct contributions of ANF types, which exhibit either high-SR, low-threshold ANF, which are activated in response to lower SPL and rapidly saturate, or low-SR, high-threshold ANF, which are recruited at higher SPL and show little or no saturation (Winter et al., 1990). CAP thresholds are insensitive to changes in low-SR ANF contributions, and only shift when high-SR ANF are affected (Bourien et al., 2014). As high-SR ANF are responsible for the shortest latencies of auditory responses at any given characteristic frequency and are suggested to define perception thresholds (Meddis, 2006; Heil et al., 2008), changes in the thresholds or latencies of an evoked CAP response might point to a contribution of such high-SR ANF.

The deletion of both MR and GR in MRGR cKO mice resulted in larger and faster ABR wave I and IV waves, with both changes occurring independently of any changes in DPOAE function. This improved neural auditory response in MRGR cKO mice was confirmed through shorter CAP latencies and improved ASSR resolution (Figure 1), indicating that compound limbic MR and GR activation might exhibit an overall negative impact on auditory temporal processing.

Interestingly, an analysis of induced, tissue-specific single MR or GR deletions revealed that the response pattern of MRGR cKO mice results from the contrasting effects of MR and GR on either auditory nerve activity and **discharge rates** (**MR cKO**) or on spike timing and **synchrony** (**GR cKO**). Thus, the lack of MR in the limbic system results in reduced IHC ribbon numbers, and specifically to a reduction of pillar-sided IHC ribbons, and reduced amplitudes of ABR wave I, while leaving ASSR responses intact (Figures 2B and 3; Figure S2B). IHC ribbons contribute through their influence on the readily-releasable vesicle pool at IHC synapses to spontaneous and evoked discharge rates of ANF, and when absent, lead to a severe reduction in ABR wave I amplitude and to deficits in onset responses and first-spike latencies (Buran et al., 2010). In the absence of IHC ribbons, the hearing threshold, dynamic range, and response precision to amplitude-modulated tones remain intact, and spreading auditory response amplitudes of ABR wave II, generated in the superior olivocochlear complex (Melcher et al., 1996), are centrally compensated (Buran et al., 2010). In MR cKO mice, the IHC ribbon numbers and ABR wave I amplitude are reduced, the ABR wave IV is enhanced, and ASSR are normal; from this we may conclude that the limbic MR affects the temporal resolving power of the auditory nerve responses through its influences on IHC ribbon integrity (Figure 6). The spatial gradient of IHC ribbon synapses provides information about postsynaptic ANF that functionally differ in SR and thresholds, if localized either on the pillar or modiolar side (Liberman, 1982; Liberman and Oliver, 1984; Merchan-Perez and Liberman, 1996). As our findings suggest a role of central MR on IHC ribbons at the pillar side, it is conceivable that under healthy conditions, central MR may improve presynaptic contact stabilization to ANF with high-SR and low thresholds that dominate more on the pillar side. It is challenging to consider for future studies if retrocochlear influences of MR activities may be realized through stress-MR-mediated effects on efferent cochlear feedback control.

In contrast, GR cKO mice showed higher-amplitude ABR waves I and IV, a shorter CAP wave I latency, reduced CAP threshold, and a better ability to detect temporally challenging, frequency-modulated tones (Figure 4), while leaving IHC ribbon numbers intact (Figure 2C). Intact IHC ribbon numbers, but altered ASSR responses, suggest that limbic GR activation exerts a “negative” top-down effect on temporal auditory processing through influencing the synchronization of spike times. Precise spike synchronization is influenced through, e.g., the fast kinetics of voltage-gated calcium channels (Ca_v_1.3) in IHC (Zidanic and Fuchs, 1995; Neef et al., 2009). These are required to ensure Ca^2+^-binding during the docking of release-ready vesicles and exocytosis (Wong et al., 2013; Rutherford et al., 2021; Johnson et al., 2019). This process critically depends on the metabolically demanding and timely retrieval of vesicles through endocytosis (Wu et al., 2014). Although the underlying details need to be determined further, limbic GR activation may provide the proper metabolic supply for precise spike synchronization. This concept is strengthened through the profound influences of CAP threshold and latency shifts in GR cKO- and MRGR cKO mice (Figures 1 and 4), that may indicate a specific vulnerability of high-SR ANF responses to limbic GR activation, as only high-SR ANF alter CAP thresholds and latencies (Bourien et al., 2014).

In addition, previous findings indirectly pointed to a GR contribution to synchronization of ANF responses. Thus, AT-induced changes in the dynamic range of auditory nerve response were weakened by GR, but not MR antagonists (Singer et al., 2018). As described in Buran et al. (2010) the dynamic range of ANF, which is defined as the range of stimulus levels over which discharge rate increases, is unaffected upon a loss of synaptic ribbons, following Bassoon deletion. This would mean that the dynamic range of the auditory nerve response is strongly affected by synchronized spike responses, but less through altered discharge rates (Buran et al., 2010). In total, the findings in GR cKO and MRGR cKO mice suggest that limbic GR activation may influence temporal auditory processing independently of discharge-rate changes of ANF, but rather through its negative impact on spike timing and synchronization of neural responses by affecting high-SR ANF processing (Figure 6). Although further studies are required to confirm the differential effect of MR and GR on discharge rate and synchronization of ANF responses, it is a striking observation that the GR function in auditory nerve processing did not correlate with blood CORT level changes. Previous studies observed limbic MR/GR functions to be the result of locally synthesized CORT on MR and GR in hippocampal cells, independently of GC produced by adrenal glands (Croft et al., 2008; Taves et al., 2011; McCann et al., 2021). Such local MR effects have previously been shown to alter e.g., social behavior or behavioral responses to novelty (McCann et al., 2021). Further studies are required to elucidate the signaling cascade by which limbic MR and GR improve or degrade auditory neural responses.

### Relation of the finding to corticosterone blood level changes

It was surprising that — despite obvious differences in the blood CORT level between GR cKO, MRGR cKO, and their littermate controls — we could not show a correlation between auditory nerve processing and the individual blood CORT level (Figure 5). As blood CORT is a central determinant of MR and GR function, we discuss reasons for this apparent disparity in depth as follows:

(i) Blood samples were taken under exactly the same conditions to avoid CORT changes. Thus, differences in GR and MR cKO CORT blood levels were not because of influences of, e.g., anesthesia level, which can dramatically change CORT (see Arnold and Langhans, 2010). We further made sure that samples were always collected by the same experimenter during the same time of the day, to minimize circadian variation of blood CORT (see Atkinson and Waddell, 1997).
(ii) Although effect sizes in male and female MR cKO, GR cKO, and MRGR cKO mice and their respective control groups were almost identical, we cannot exclude gender-related effects of blood CORT on auditory nerve function.
(iii) As differences in the blood CORT level were only observed in GR cKO and MRGR cKO, but not MR cKO mice, significant effects of central MR on auditory nerve responses are unlikely to be controlled by plasma CORT levels.
(iv) High levels of basal CORT reportedly impaired auditory nerve responses (Singer et al., 2018). In the present study, GR cKO mice had the largest auditory nerve responses, but at the same time the highest levels of basal blood CORT. In this group of animals, we saw an opposite relation between mean CORT and ABR wave I amplitude, which is another hint that CORT itself is not responsible for the reduced inhibition of auditory nerve sensitivity.

However, in many disorders, HPA-axis activity abnormalities are not evident in baseline blood plasma CORT levels, but are observed in the presence of an acute stressor. Further studies are required to test the extent to which stress levels in MR, GR, or MRGR cKO are different under acute stress and if such changes affect the processing of sound. So far, our data allow us to conclude that the changes in peripheral auditory processing and IHC ribbon synapses in MR cKO, GR cKO, and MRGR cKO are not directly related to changes in the blood CORT level. Mechanisms underlying this top-down signaling from central MR/GR to the auditory periphery remain, however, unclear at present.

Besides CORT itself, other hormones influence the body’s responses after exposure to stressful events. Corticotropin releasing hormone (**CRH**) is highly expressed and widely distributed in neurons of the CNS. A large amount of CRH is synthesized in the paraventricular nucleus, and its release stimulates the pituitary gland to produce and secrete adrenocorticotropin (Richard and Lopez, 2013). Beside its modulation of the HPA-axis, CRH is responsible for food intake and energy expenditure (Richard and Lopez, 2013), and in several species CRH also controls the HPA-axis by inducing the secretion of thyroid-stimulating hormone (De Groef et al., 2006) and thereby possibly plays a key role in the endocrine regulation of life-stage transitions. Effects of CRH on ABR wave I are unclear, although it has previously been shown that CRH plays a role in cochlear hearing sensitivity and noise vulnerability (Vetter, 2015; Graham et al., 2010, 2011) and is expressed during the development of hair-cell innervation (Graham and Vetter, 2011). Further quantification of CRH blood level and its local expression in the cochlea should help clarify the role of CRH in ANF signaling in MR cKO, GR cKO, and MRGR cKO mice.

Previous studies observed limbic MR/GR activation in response to locally synthesized CORT in hippocampal cells, independently of GC produced by adrenal glands (Croft et al., 2008; Taves et al., 2011; McCann et al., 2021). Moreover, local MR activation has previously been shown to alter, e.g., social behavior or behavioral responses to novelty (McCann et al., 2021). CA2 pyramidal cells are further distinguished from neighboring CA1 and CA3 pyramidal cells in that they exhibit a unique pattern of gene expression that permits tight regulation of synaptic plasticity of Schaffer’s collateral synapses (McCann et al., 2021), and confers sensitivity to the social neuropeptides oxytocin and vasopressin (Pagani et al., 2015; Raam et al., 2017). Vasopressin and oxytocin are expressed not only in the limbic forebrain (Cilz et al., 2019) but also in the cochlea (Reuss et al., 2009). Oxytocin also stimulates soluble guanylate cyclase and increases intracellular cGMP (Porzionato et al., 2010; Conrad et al., 1993), the signaling cascade that was shown to act on auditory nerve processing (Chumak et al., 2016; Marchetta et al., 2020, 2021).

Alternatively, MR and GR forebrain activities may exhibit retrocochlear top-down effects to the periphery through e.g., the olivocochlear efferent feedback system, which has been shown to be activated by selective attention (for review see (Lopez-Poveda, 2018)). Here, one very important modulator is the lateral olivocochlear system (Guinan, 2006), where among other neurotransmitters dopamine plays a crucial role (for review see (Lopez-Poveda, 2018)). In the cochlea, dopamine acts at a postsynaptic level through axodendritic auditory nerve terminal, where it tonically decreased CAP wave I amplitude and prolonged latency when the cochlea was perfused with dopamine (Ruel et al., 2001). It is thus challenging to consider a contrasting modification of dopamine mediated tonic inhibition as a target of either GR or MR triggered retrocochlear feedback.

### Role of central/limbic MR in perceptual auditory object formation

In conclusion, we suggest that MR in the limbic brain or GR in central brain regions, both shown to optimize stress-coping (de Kloet et al., 2019), control top-down signaling to the auditory system by improving or weakening IHC synapse processing.

Numerous studies that describe positive effects of acute or low CORT levels on hearing (Meltser and Canlon, 2011; Canlon et al., 2013) should be reconsidered in the light of our present findings on limbic MR activities. This includes the acute and subchronic administration of hydrocortisone, which was shown to transiently enhance the amplitude of auditory evoked potentials in normal subjects (Ashton et al., 2000; Born et al., 1989). It also includes acute restraint stress, which was observed to improve sound-induced responses in the auditory cortex (Ma et al., 2015), and finally low-dose CORT effects that enhanced the amplitude of auditory evoked potentials recorded from electrodes placed in the CA3 region of the hippocampus (Maxwell et al., 2006). In all cases, limbic MR activation explains these findings because of its direct impact on peripheral auditory processing. Limbic MR activities might be a most attractive key top-down signature linking limbic and auditory neural circuits that are causatively involved in improved hearing, speech discrimination, or communications skills (Jauset-Berrocal and Soria-Urios, 2018; Sihvonen et al., 2017; Sinha et al., 2011; Schaffert et al., 2019; Micheyl et al., 2006). A reduced expression of MR could be linked to neurodevelopmental disorders, such as autism spectrum disorder (Patel et al., 2016) and a human genome mutation leading to a stop-gain alteration of MR protein was found in three brothers with autism (Cukier et al., 2020). Autism spectrum disorder has been hypothesized to be linked with auditory processing deficits in humans (Foss-Feig et al., 2017; Beers et al., 2014) and animal models (Eckert et al., 2021; Truong et al., 2015).

On the other hand, numerous studies point to high CORT or chronic stress as diminishing auditory gating (Maxwell et al., 2006; Stevens et al., 2001; Elling et al., 2011; White et al., 2005; Ma et al., 2017), or to reduced auditory responsiveness following acoustic trauma-induced stress (Ryan et al., 2016; Matt et al., 2018), post-traumatic stress, or chronic stress (MacGregor et al., 2020; Turner et al., 2019; Kreuzer et al., 2014; Mazurek et al., 2019). These studies may now be reconsidered in the context of a possibly direct impact of limbic GC/GR signaling on spike-timing precision and synchronization of neural auditory responses. The close relationship between distress and tinnitus (Boecking et al., 2021; Elarbed et al., 2021; Park et al., 2020; van Munster et al., 2020; Clifford et al., 2019), and the increasing evidence suggesting a role of high-SR ANF processing deficits in tinnitus (Knipper et al., 2013), must be adapted to reflect the herein described limbic GR effects on cochlear function. Interestingly it was shown that patients suffering from a hyperfunctioning pituitary tumor (Cushing Disease) have an increased risk for hearing impairments as comorbidities (Kuan et al., 2017). On the other hand, adrenal cortical insufficiency led to lower hearing thresholds and higher hearing sensitivity in patients, such as in our GR cKO mice (Henkin et al., 1967).

Within this context, the present findings should guide consideration of limbic MR and GR effects on auditory processing, possibly being one of the enigmatic contributors to perceptual auditory object recognition. Although all acoustic input in the environment is detected in multiple “streams,” attention can be laid on each of the streams selectively and is used when a person follows e.g., a musical instrument in the middle of an orchestra (Pressnitzer et al., 2008). Such streaming allows the suppression of responses to unimportant auditory cues in a sound mixture, and thereby influences auditory perception, understanding, and behavioral responses in hearing function in an everyday setting. For object recognition, natural auditory environments or “scenes” require listening to sounds at different time points and frequencies to match the incoming auditory input with stored central information to, e.g., isolate and match voices in a crowded environment to previously memorized information and thus recognize the person (Cope et al., 2017). This process of auditory perception has been found to use “precognitive” subcortical processing information (Michie et al., 2016; Perez-Gonzalez and Malmierca, 2014; Antunes and Malmierca, 2021), possible tuned as low as the cochlea (Pressnitzer et al., 2008). The key signature that bridges the “central hearing” and the “peripheral hearing” to extract the information in “scenes” is currently missing (Johnson et al., 2021). As MR plays a role in selective attention (Cornelisse et al., 2011), we may hypothesize that recruitment of limbic MR activities could be a possible candidate mediator to bridge central processing with peripheral processing during the streaming process.

Overall, the roles that MR activation is predicted to play in attention, decision-making, and empathy (Wingenfeld and Otte, 2019; Joels, 2018; Chumak et al., 2016), and that GR activation is predicted to play in memory deficits, cognitive decline, and psychopathologies including Alzheimer’s disease (Finsterwald and Alberini, 2014; Ouanes and Popp, 2019; Johnson et al., 2021), make MR and GR most attractive candidates for positive and negative “precognitive” cochlear processing during auditory perception and auditory cognitive dysfunction (Johnson et al., 2021).

### Limitations of the study

The present study demonstrates that central/limbic deletion of MR and/or GR leads to changes in peripheral auditory processing and IHC ribbon synapses. This indicates a defective top-down signaling in the auditory system of MR cKO, GR cKO, and MRGR cKO. With the experimental setting used in the present study, we found no correlation of blood CORT levels with the observed auditory phenotypes. Future studies thus might expose a possible spatiotemporal window of blood CORT not yet identified, through which MR/GR signaling activities alter cochlear nerve processing.

## STAR★Methods

### Key resources table

**Table:** 

REAGENT or RESOURCE	SOURCE	IDENTIFIER
**Antibodies**		

Rabbit Polyclonal C-terminal-binding protein 2 (CtBP2)/RIBEYE	ARP American Research Products, Inc™, Waltham, MA, USA	Cat#10-P1554
Rabbit Polyclonal Vesicular Glutamate Transporter 3	Synaptic Systems, Göttingen, Germany	Cat#135203 RRID:AB_887886
Mouse Monoclonal Mineralocorticoid Receptor	Thermo Fisher, Rockford, IL, USA	Cat#H10E4C9F/MA1-620 RRID:AB_2298880
Mouse Monoclonal Glucocorticoid Receptor	Thermo Fisher, Rockford, IL, USA	Cat#BUGR2/MA1-510 RRID:AB_2811764
Secondary Antibody Cy3	Jackson Immuno Research Laboratories, West Grove PA, USA	Cat#111-166-046 RRID:AB_2338009
Secondary Antibody Alexa 488	Molecular Probes , Eugene, OR, USA	Cat#A11001

**Chemicals, peptides, and recombinant proteins**		

Tamoxifen	Sigma-Aldrich	SKU#T5648-1G
Fentanyl	Fentanyl-Hameln, Hameln Pharma plus, Hameln, Germany	PZN#06143427
Midazolam	Midazolam-hameln®; Hameln Pharma plus, Hameln, Germany	PZN#4467367
Medetomidin	Sedator®; Albrecht, Aulendorf, Germany	PZN#1901022
Atropine sulphate	B. Braun, Melsungen, Germany	PZN#00648037
Ampuwa	Fresenius KABI, Bad Homburg, Germany	PZN#10333435
Naloxon	Naloxon-hameln®; Hameln Pharma plus, Hameln, Germany	PZN#04464535
Flumazenil	Flumazenil®; Fresenius KABI, Bad Homburg, Germany	PZN#04952364
Atipazemol	Antisedan®; VETOQUINOL GmbH, Ravensburg, Germany	GTIN#05012674902110
Xylocain 2%	AstraZeneca, Wedel, Germany	PZN# 01138002
sunflower oil	Sigma-Aldrich	SKU# S5007-250ML

**Critical commercial assays**		

CORT ELISA kit	Enzo Life Sciences Inc., Farmingdale, NY, USA	Cat#ADI-901-097

**Deposited data**		

Raw and analyzed data	This paper	N/A

**Experimental models: Organisms/strains**		

CaMKIIαCre^ERT2^ mice: C57BL6/N-TgN(CaMKIIaERT2-cre)1743/2Gsc	Prof. Günther Schütz (DKFZ, Molecular Biology of the Cell I, Heidelberg, Germany)	N/A
MR cKO mice: C57BL6/N-TgH(MRflox)1101/2Gsc x TgN(CaMKIIaERT2-cre)1743/2Gsc	Prof. Günther Schütz (DKFZ, Molecular Biology of the Cell I, Heidelberg, Germany)	N/A
GR cKO mice: C57BL6/N-TgH(GRflox)1103/2Gsc x TgN(CaMKIIaERT2-cre)1743/2Gsc	Prof. Günther Schütz (DKFZ, Molecular Biology of the Cell I, Heidelberg, Germany)	N/A
MRGR cKO mice: C57BL6/N-TgH(GRflox)1103/2Gsc x TgH(MRflox)1101/2Gsc x TgN(CaMKIIaERT2-cre)1743/2Gsc	Prof. Günther Schütz (DKFZ, Molecular Biology of the Cell I, Heidelberg, Germany)	N/A
Rosa^tdTomato^ reporter mouse	Prof. Hubert Löwenheim from the Tübingen Hearing Research Centre (THRC)	N/A

**Software and algorithms**		

GraphPad PRISM Version 5.01	GraphPad Software, Inc.	www.graphpad.com
CorelDRAW Version 15.2.0.695	Corel Corporation	www.corel.com
Microsoft Excel	Microsoft Corporation	www.microsoft.com
PEAK.exe	University of Tübingen	N/A
CAP.exe	University of Tübingen	N/A

### Resource availability

#### Lead contact

Further information and requests for resources and reagents should be directed to and will be fulfilled by the lead contact, Marlies Knipper (marlies.knipper@uni-tuebingen.de).

#### Materials availability

This study did not generate new, unique reagents.

### Experimental model and subject details

In the present study, three tamoxifen-inducible conditional knock-out mouse lines were studied, in which MR and GR or either MR or GR are deleted, mainly in the forebrain. To generate the MRGR cKO, MR cKO and GR cKO mice and corresponding control animals, three different mouse lines were used. We received from Prof. Günther Schütz (DKFZ, Molecular Biology of the Cell I, Heidelberg, Germany) homozygous floxed MR and GR mouse lines (Berger et al., 2006; Erdmann et al., 2007), in which the exon 3 of either *Mr* or *Gr* is flanked by loxP sites. These lines were crossed in our laboratory to obtain a homozygous MRGRflox line. These three lines were than bred with a CaMKIIα Cre^ERT2^ line (Erdmann et al., 2007) (kindly provided by Prof. Günther Schütz) in which the Cre-Recombinase is expressed under the CaMKIIα promoter after Tamoxifen (**TMX**) injection. In brief, after confirmation of a normal hearing function, mice received an intraperitoneal injection of 1 mg TMX in 100 μl TMX-solution (Sigma-Aldrich, T-5648, Munich) twice a day on 5 consecutive days at the age of approximately 8 weeks. For the solution, 50 mg TMX was dissolved in 500 μl Ethanol abs. (Merck, Darmstadt) and mixed with 4.5 ml sunflower oil (Sigma-Aldrich, S-5007). After the last injection, the animals were allowed to recover in their home cages for four weeks before the experiments started. The Cre-recombinase leads to the excision of exon 3 of *Mr* and *Gr* or either *Mr* or *Gr* in cells where CaMKIIα is expressed. To verify the deletion pattern of MR and GR, CaMKIIαCre^ERT2^ transgenic mice (Erdmann et al., 2007), were crossed with a Rosa^tdTomato^ reporter mouse line (Madisen et al., 2010), kindly provided by Prof. Hubert Löwenheim from the Tübingen Hearing Research Centre (THRC). For all transgenic mouse lines, homozygous floxed Cre-negative littermates that also received TMX were used as controls. For all lines, mice of both sexes aged between 1.8 and 8.4 months were used. The genetic status of all mouse lines was confirmed by genotyping using gene-specific PCR protocols.

Mice were housed in the animal facility of the ENT University Hospital of Tübingen and had access to water and food pellets *ad libitum*. They were housed alone or in groups of 2 to 5. Females and single males had a wooden house or tunnel in their cages. The dark-light cycle was 12-12 h, with a light period from 6 am to 6 pm summer time. Humidity was 55 (±5) % and temperature 21.5 (±1)°C. The weight of the animals was controlled on every experimental day. The average noise level in the animal facility was below 50 – 60 dB SPL.

Animal care, procedures, and experimental protocols corresponded to national and institutional guidelines and were reviewed and approved by the University of Tübingen Veterinary Care Unit and the Animal Care and Ethics Committee of the regional board of the Federal State Government of Baden-Württemberg, Germany. All experiments were performed according to the European Union Directive for the protection of animals used for experimental and other scientific purposes (2010/63/EU). Mice were kept according to the national guidelines for animal care in a specifically pathogen-free animal facility.

### Method details

#### Hearing measurements

Hearing function was studied before and after TMX-induction by measuring Distortion Product Otoacoustic Emission (**DPOAE**), Auditory Brainstem Responses (**ABR**) and Elelctrocochleographic Recordings in a soundproof chamber (IAC 400-A, Industrial Acoustics Company GmbH, Niederkrüchten).

Mice were anesthetized with an intraperitoneal injection of a mixture of Fentanyl (Fentanyl-Hameln, Hameln Pharma plus, Hameln, Germany), Midazolam (Midazolam-hameln®; Hameln Pharma plus, Hameln, Germany), Medetomidin (Sedator®; Albrecht, Aulendorf, Germany) and atropine sulfate (B. Braun, Melsungen, Germany) diluted with water ad. inj. (Ampuwa, Fresenius KABI, Bad Homburg, Germany) to an injection volume of 10 ml per kg bodyweight. Additional doses of anesthetics were administered if needed. The anesthesia was antagonized after the measurements by a subcutaneously administered mixture of Naloxon (Naloxon-hameln®; Hameln Pharma plus, Hameln, Germany), Flumazenil (Flumazenil®; Fresenius KABI, Bad Homburg, Germany), and Atipazemol (Antisedan®; VETOQUINOL GmbH, Ravensburg, Germany) diluted with water ad. inj. (Ampuwa, Fresenius KABI, Bad Homburg, Germany) to an injection volume of 10 ml/kg.

#### DPOAE

For DPOAE measurements, the anaesthetized mice lay on a pre-warmed resting pad (37°C) in the soundproof chamber and an acoustic coupler was carefully placed in the ear canal. The cubic 2f1 - f2 DPOAE was measured for frequencies (f) f2 = 1.24 × f1 and levels (L) L2 = L1 - 10 dB using a sensitive microphone inside the coupler (MK231, Microtech Gefell, Gefell, Germany, Preamplifier B&K 2669C, Bruel & Kjaer, Naerum, Denmark). Stimuli pair presented contained frequencies between f2 = 4.0 to 32.0 kHz with L2 either constantly at 50 dB SPL (DP-gram) or increasing from -5 to 65 dB SPL in 5 dB steps (I/O growth function).

#### ABR

The anaesthetized mice lay on a pre-warmed resting pad (37°C) in the soundproof chamber. ABR in anesthetized mice were evoked by short-duration sound stimuli with the same stimulus parameters for all groups of KO and control animals. They represent the summed activity of neurons in distinct anatomical structures along the ascending auditory pathway recorded from subcutaneous cranial electrodes. A microphone (Bruel & Kjaer 4191, Naerum, Denmark) was used to calibrate and record the acoustic stimuli. ABR thresholds were elicited with click (100 microsecond duration with an FFT mean of 5.4 kHz), noise-burst (1 ms duration, FFT mean of 7.9 kHz), or pure-tone stimuli (3 ms duration, including 1 ms cosine squared rise and fall envelope, 2–32 kHz). The stimulus level was increased stepwise from 10 to 100 dB SPL in 5 dB steps. Stimuli were generated with an I-O-card (PCI-6052E, PCI-6251, or PCIe-6259, National Instruments, Austin, Texas, USA) in an IBM compatible computer. The SPL of the stimuli was modulated by custom-made amplifier and attenuator systems (Wulf Elektronik, Frankfurt). The measured signals were band-pass filtered from 200 Hz to 5 kHz (F1, 6-pole Butterworth hardware Filter, Wulf Elektronik, Frankfurt) and amplified by 100,000. The analog/digital **(A/D)** rate was 20 kHz. Each stimulus had a recording interval of 16 ms and was directly repeated and averaged up to 512 times (256 for pure-tone stimuli).

#### Auditory steady-state responses

The response on amplitude modulation was tested on the ear with the lower click- and noise-evoked threshold directly after finishing the standard ABR protocol, with similar electrode positions. Auditory steady-state responses (**ASSR**) were measured with amplitude-modulated sinusoidal stimuli (carrier frequency 11.31 kHz). For the modulation depth function, stimuli were amplitude modulated with modulation depth varying from 0% (unmodulated) to 100% (maximal modulation) and 512 Hz modulation frequency, at 40 dB relative to threshold. For the growth function, the stimuli, modulated 100% with 512 Hz, were presented between 0 and 60 dB relative to threshold in 5 dB steps. For the transfer function, stimuli were modulated with a frequency between 64 and 2048 Hz at 100% modulation depth and 40 dB relative to threshold.

#### Electrocochleographic recordings

We studied electrical potentials of auditory nerve fibers (**ANF**) by electrocochleography in living anaesthetized mice. The mice were anesthetized as described above, 20-40 μl Xylocain 2% (AstraZeneca, Wedel, Germany) was applied subcutaneously at sites of surgical incisions and the mice were laid on a pre-warmed resting pad (37°C). The bony auditory bulla was exposed by cutting the skin behind the ear and carefully moving muscles, nerves, and connective tissues beside. A small hole (0.6 mm diameter) was drilled into the bulla, and the round-window niche of the cochlea visualized. A silver wire electrode insulated by varnish and silicone and ending in a small silver bead was placed within the niche. The skin above the ear was closed and the mouse placed in the sound-attenuating booth in front of a loudspeaker for recording. Compound action potential (**CAP**) threshold responses from the auditory nerves were measured by stimulation with short tone pips (3 ms duration including 1 ms on- and off-ramp cos-square shaped, 32-96 repetitions with stimulus interval 16 ms and alternating polarity) presented with 5 dB 12 incremental steps from 0-100 between 2 and 34 kHz. Electrical potentials were amplified (80 dB) and filtered between 0.2 and 5 kHz before being sampled at 20 kHz A/D rate, averaged, and saved to file. Thresholds were determined from individual ears from averaged waveform responses as the lowest SPL, resulting in a signal visually distinguishable from noise.

For the CAP latency, electrical responses were recorded for 100 μs click stimuli of 0 to 100 dB SPL. Responses were amplified, filtered (DC, 50 kHz low pass), sampled at 100 kHz A/D rate, and averaged for 64 repetitions (ISI 50 ms). For CAP input-output analysis, the averaged waveform was manually inspected for the first negative amplitude deflection after stimulus onset. The latency of the CAP is registered for each stimulus intensity for each individual ear and the resulting growth function averaged and presented as the mean and SEM.

#### Tissue preparation

The mice were euthanized by exposure to CO_2_. After decapitation the skull was opened and the complete brain was removed. The residual skull was cut in half and the cochleae were removed from the temporal bone under the microscope.

For cochlear whole-mounts the cochleae were isolated, fixed by immersion in 4% paraformaldehyde, 125 mM sucrose in 100 mM phosphate buffered saline (pH 7.4) for 15 min on ice and then dissected for whole-mounts. For cochlear cross-sections, the cochleae were fixated in 2% paraformaldehyde for 2h at 4°C on a rotating wheel, decalcified until the bone was soft and stored in Sucrose-Hank’s solution (4°C) over night. Afterwards the cochleae were embedded in Tissue-Tek, frozen at -80°C and sliced in 10 μm sections, using a Cryostat (Leica Cryostat 1720 Digital Leica, Wetzlar, Germany).

Brains were fixed by immersion for 48 h in 2 % paraformaldehyde (exchange of fixative solution after 24 h) and then stored in 0.4 % paraformaldehyde until embedded in 4% agarose. Brains were cut in 60 μm slices with a vibratome (Leica VT 1000S) and stored at -20°C in cryoprotectant (mix 150 g of sucrose in 200 ml 1× phosphate buffer saline (**PBS**) and 150 ml ethylene glycol) until used for immunohistochemistry.

#### Immunohistochemistry and ribbon counting

Object slides with cochlear sections or whole-mount preparations were thaw at room temperature for 30 min. After permeabilization for 10 min, the tissue was rinsed with PBS and blocking solution was given to each slice for 30 min. The primary antibody was diluted in reaction buffer and applied to the object slides. After incubation overnight at 4°C and 3x washing in PBS, the secondary antibody, which was diluted in reaction buffer was pipet and incubated for 1h at room temperature. After 3x rinsing in PBS, the cochlear tissue was covered using Vectashield mounting medium with DAPI.

For free-floating brain immunohistochemistry, brain slices were taken out of the cryosolution and transferred into 1× PBS. After 2x washing with PBS for 15 min, the tissue was permeabilized and blocked for 30 min in 3 % BSA containing 0.2 % Triton-X 100. Primary antibodies were diluted in 0.5–1.5 % BSA, containing 0.1 % Triton-X 100 for incubation at 4°C overnight. The slices were washed 3x for 15 min in 1× PBS before 1h incubation at room temperature with secondary antibodies (diluted in 0.5–1.5 % BSA, containing 0.1 % Triton-X 100). The slices were washed 3x for 15 min in 1× PBS, transferred to object slides and mounted with Vectashield mounting medium with DAPI.

Antibodies against C-terminal-binding protein 2 (CtBP2)/RIBEYE (rabbit, diluted 1:1500; ARP American Research Products, Inc™, Waltham, MA, USA), VGlut3, (rabbit, diluted 1:1500; Synaptic Systems, Göttingen, Germany), MR (mouse, diluted 1:500; Thermo Fisher, Rockford, IL, USA) or GR (mouse, diluted 1:500; Thermo Fisher, Rockford, IL, USA) were used. Primary antibodies were detected using appropriate secondary antibodies Cy3 (1:1500, Jackson Immuno Research Laboratories, West Grove PA, USA) and Alexa 488 (1:500, Molecular Probes, Eugene, OR, USA).

All samples were viewed using an Olympus BX61 microscope (Olympus, Hamburg, Germany) equipped with an X-Cite epifluorescence illumination. Images were acquired using an Olympus XM10 CCD monochrome camera and analyzed with CellSens Dimension software (OSIS GmbH, Münster, Germany). To increase spatial resolution, slices were imaged over a distance of ∼15 μm within an image-stack along the z-axis (z-stack), followed by 3-dimensional deconvolution using CellSens Dimension’s built-in algorithm.

Cross-sections from the apical, medial, mid-basal and basal half-turn of the mouse organ of Corti correspond to frequency ranges of 2–7 kHz (apical), 7–16 kHz (medial), 16 –36 kHz (mid-basal) and 36 –70 kHz (basal) as estimated from place frequency maps (Muller et al., 2005).

For the ribbon gradient analysis, at least two deconvoluted pictures of a z-stack, rotated to a proper orientation for pillar vs. modiolar IHC sides were analyzed per animal. A line was drawn through the center of the IHC by two blinded persons and modiolar and pillar ribbons were counted. The amount of pillar/modiolar ribbons was calculated in %, averaged from all pictures per animal and both person’s judgements. and presented as the mean and SD and analyzed by unpaired Student’s t-test with α = 0.05 (GraphPad Prism).

#### Corticosterone analysis

Corticosterone (**CORT**) concentration was measured in venous blood samples that were collected into heparin-coatedmicrovettes (Sarsted) directly after the onset of anesthesia for hearing measurements (i.e. < 5 min after handling and injection) in the time window between 9 and 12 am. Each blood sample was around 50 - 70μl. After collection, the sample was centrifuged at 1800 x *g* for 5 min and the plasma was pipetted to 1.5 ml Eppendorf tubes and stored at – 80°C. The analysis was assessed by using CORT ELISA kit (Catalog Nr. ADI-901-097) from Enzo Life Sciences Inc. (Farmingdale, NY, USA), following the manufacturers protocol. The plates were read with an optima FLUOstar microplate reader at 405 nm.

### Quantification and statistical analysis

All statistical information and n numbers can be found in the results section and in Table 1. In figures, significance and a trend for significance is indicated by asterisks ((∗) *p* < 0.1, ∗ *p* < 0.05, ∗∗ *p* < 0.01, ∗∗∗ *p* < 0.001). n.s. denotes non-significant results (*p* ≥ 0.05). The p-values of the 2-way ANOVAs refer to the main effect of the genotype.

**Table 1:** Detailed statistical information

Fig. No	Context	Statistical test	Test value	p value	Bonferroni *post hoc* test with p value		*n*-number
1B	Click	unpaired two-tailed Student’s *t* test	t(50) = 0.620	p = 0.538	-	-	WT: n = 12/24, KO: n = 14/28 mice/ears
	Noise		t(50) = 0.451	p = 0.653			
	f-ABR	two-way ANOVA	F(1,5) = 1.772	p = 0.186	-	-	WT: n = 10, KO: n = 11 mice/ears
1C	DPOAE	two-way ANOVA	F(1,5) = 1.772	p = 0.186	-	-	WT: n = 5/10, KO: n = 8/16 mice/ears
1D	CAP	two-way ANOVA	F(1,16) = 0.368	p = 0.5443	-	-	WT: n = 5/9, KO: n = 7/13 mice/ears
1F	ABR wave I	two-way ANOVA	F(1,17) = 10.80	p = 0.0011	-	-	WT: n = 12/24, KO: n = 15/30 mice/ears
	ABR wave IV		F(1,17) = 31.62	p < 0.0001	-	-	WT: n = 12/24, KO: n = 14/28 mice/ears
1G	Latency	two-way ANOVA	F(1,620) = 70.94	p = 0.0022	-	-	WT: n = 6/11, KO: n = 7/13 mice/ears
1H	AMD	two-way ANOVA	F(1,572) = 11.38	p = 0.0008			WT: n = 19, KO: n = 18 mice/ears
1I	AMG		F(1,688) = 5.210	p = 0.023			WT: n = 18, KO: n = 20 mice/ears
1J	AMT		F(1,473) = 14.37	p = 0.0002	-	-	n = 19 mice/ears
2A	MRGR ribbons	two-way ANOVA	F(1,2) = 41.10	p < 0.0001	apical	p > 0.05	WT: n = 19/9, KO: n = 18/9 IHC/mice
					medial	p < 0.001	
					mid-basal	p < 0.001	
2B	MR ribbons	two-way ANOVA	F(1,2) = 44.25	p < 0.0001	apical	p > 0.05	WT: n = 12/5, KO: 14/5 IHC/mice
					medial	p < 0.001	
					mid-basal	p < 0.001	
2C	GR ribbons	two-way ANOVA	F(1,2) = 6.603	p *= 0*.*0125*	apical	p > 0.05	WT: n = 10/5, KO: n = 14/6 IHCs/mice
					medial	p > 0.05	
					mid-basal	p > 0.05	
3A	ABR wave I	two-way ANOVA	F(1,18) = 19.40	p < 0.0001	-	-	WT: n = 8/16, KO: n = 8/16 mice/ears
	ABR wave IV		F(1,17) = 6.991	p = 0.0085	-	-	WT: n = 8/16, KO: n = 8/16 mice/ears
3B	CAP	two-way ANOVA	F(1,16) = 14.92	p = 0.0001	-	-	n = 7/14 mice/ears
	Latency	two-way ANOVA	F(1,733) = 5.57	p = 0.0186	-	-	WT: n = 7/14, KO: n = 6/11 mice/ears
3C	AMD	two-way ANOVA	F(1,701) = 0.14	p = 0.999	-	-	WT: n = 24, KO: n = 21 mice/ears
	AMG		F(1,955) = 0.01	p = 1.000			WT: n = 24, KO: n = 22 mice/ears
	AMT		F(1,583) = 0.02	p = 0.8905	-	-	WT: n = 24, KO: n = 22 mice/ears
4A	ABR wave I	two-way ANOVA	F(1,17) = 45.83	p < 0.0001	-	-	WT: n = 7/14, KO: n = 8/16 mice/ears
	ABR wave IV		F(1,17) = 57.51	p < 0.0001	-	-	WT: n = 7/14, KO: n = 8/16 mice/ears
4B	CAP	two-way ANOVA	F(1,16) = 14.60	p = 0.0001	-	-	WT: n = 8/15, KO: n = 9/18 mice/ears
	Latency	two-way ANOVA	F(11,043) = 14.04	p = 0.0002	-	-	WT: n = 10/20, KO: n = 10/19 mice/ears
4C	AMD	two-way ANOVA	F(1,780) = 15.56	p < 0.0001	-	-	WT: n = 26, KO: n = 24 mice/ears
	AMG		F(11,025) = 34.58	p < 0.0001			WT: n = 30, KO: n = 28 mice/ears
	AMT		F(1,636) = 9.99	p = 0.0017	-	-	WT: n = 26, KO: n = 24 mice/ears
5B	CORT MR	unpaired two-tailed Student’s *t* test	t(28) = 90.9285	p = 0.3611	-	-	WT: n = 16, KO: n = 14 mice
	CORT GR		t(28) = 1.884	p = 0.070			WT: n = 14, KO: n = 16 mice
	CORT MRGR		t(24) = 2.905	p = 0.0078	-	-	WT: n = 12, KO: n = 14 mice
5C	corr. MR	linear regression	WT: R^2^ = 0.033	p = 0.5329	-	-	WT: n = 14, KO: n = 13 mice
			KO: R^2^ = 0.001	p = 0.9322			
	corr. GR		WT: R^2^ = 0.012	p = 0.7802			n = 9 mice
			KO: R^2^ = 0.0001	p = 0.9723			
	corr. MRGR		WT: R^2^ = 0.141	p = 0.2540			WT: n = 11, KO: n = 17 mice
			KO: R^2^ = 0.007	p = 0.7574	-	-	
S2B	MRGR	unpaired two-tailed Student’s *t* test	t(15) = 3.57	p = 0.0028			WT: n = 9, KO: n = 8 mice
	MR		t(10) = 2.38	p = 0.0387			n = 6 mice
	GR		t(8) = 0.79	p = 0.4551			n = 5 mice
S3A	Click	unpaired two-tailed Student’s *t* test	t(56) = 0.07	p = 0.944			WT: n = 14/28, KO: n = 15/30 mice/ears
	Noise		t(56) = 0.64	p = 0.519	-	-	
	f-ABR	two-way ANOVA	F(1,8) = 0.045	p = 0.832			WT: n = 14, KO: n = 15 mice
S3B	DPOAE	two-way ANOVA	F(1,5) = 4.030	p = 0.455	-	-	WT: n = 5/10, KO: n = 8/16 mice/ears
S3C	Click	unpaired two-tailed Student’s *t* test	t(58) = 1.398	p = 0.168	-	-	WT: n = 14/28, KO: n = 16/32 mice/ears
	Noise		t(58) = 0.124	p = 0.902			
	f-ABR	two-way ANOVA	F(1,8) = 0.078	p = 0.781	-	-	WT: n = 13, KO: n = 16 mice
S3D	DPOAE	two-way ANOVA	F(1,5) = 0.182	p = 0.670	-	-	WT: n = 14/28, KO: n = 16/32 mice/ears

#### Hearing measurements

##### DPOAE

For analysis, the respective thresholds for the single frequencies were identified. The criteria for reaching the threshold, were a signal level above -15 dB SPL, 5 dB above noise level, and as part of an increasing function. Data were presented as the mean and SEM and analyzed by 2-way ANOVA with α = 0.05, followed by Bonferroni *post hoc* test (GraphPad Prism).

##### ABR

The threshold for all click, noise, and pure-tone ABR measurements were manually defined as the lowest sound level at which a clear signal could be discriminated from the baseline. Data are shown as the mean ± SEM. Click- and noise-evoked ABR measurements were compared between genotypes by an unpaired Student’s t-test with α = 0.05. F-ABR measurements were group analyzed by 2-way ANOVA with α = 0.05 followed by Bonferroni *post hoc* test (GraphPad Prism).

Supra-threshold click-evoked ABR waveforms were analyzed for consecutive amplitude deflections (waves), with each wave consisting of a starting negative (n) peak and the following positive (p) peak. Two peak classes were selected: (1) early peaks (wave I: In − Ip at 1.2-1.8 ms) interpreted as the sum of the first stimulus-related action potential within the auditory nerve, and (2) late peaks (wave IV: IVn − IVp at 4.1-4.9 ms), the response from the auditory midbrain. All ABR wave supra-threshold amplitude growth functions were calculated for increasing stimulus levels with reference to the ABR thresholds (from 0 to a maximum of 80 dB above threshold). For Figure 5 the ABR response amplitude size was calculated by averaging the three maximal amplitude values of the individual ears’ supra-threshold amplitude growth function. In Figure 5 for each animal the mean of both ears’ ABR wave I size was correlated with the CORT level of the animal. Data were shown as the mean ± SEM and analyzed by 2-way ANOVA with α = 0.05 followed by Bonferroni *post hoc* test (GraphPad Prism).

##### ASSR

For analysis, a fast Fourier transform (FFT) of the response was calculated. From the FFT, the spectral amplitude at the modulation frequency was extracted, along with the first five harmonics. Additionally, from the FFT, the noise level from the neighboring frequency spectral amplitude (± 4 Hz) was extracted. From this, the signal above noise (μV) was calculated. Data were shown as the mean ± SEM and analyzed by 2-way ANOVA with α = 0.05 followed by Bonferroni *post hoc* test (GraphPad Prism).

##### Electrocochleographic recordings

Thresholds were determined from individual ears from averaged waveform responses as the lowest SPL resulting in a signal visually distinguishable from noise. Data were analyzed by 2-way ANOVA with α = 0.05 (GraphPad Prism). For CAP input-output analysis, the averaged waveform was manually inspected for the first negative amplitude deflection after stimulus onset. The latency of the CAP was registered for each stimulus intensity for each individual ear, and the resulting growth function averaged and presented as the mean and SEM. Data were analyzed by 2-way ANOVA with α = 0.05 followed by Bonferroni *post hoc* test (GraphPad Prism).

#### Ribbon counting

Ribbons are shown as average ribbon number per IHC ± SEM. Statistical analysis was performed using 2-way ANOVA with α = 0.05 followed by Bonferroni *post hoc* test (GraphPad Prism). The amount of pillar/modiolar ribbons was presented as the mean and SD and analyzed by unpaired Student’s t-test with α = 0.05 (GraphPad Prism).

#### Corticosterone analysis

Calculation of the CORT levels were performed using an online data analysis tool (myassays.com). Finally, CORT levels were averaged per genotype and presented as the mean ± SEM. Statistical analysis was performed by unpaired Student’s t-test with α = 0.05 (GraphPad Prism).

## Data Availability

All data reported in this paper will be shared by the lead contact upon request. This paper does not report original code. Any additional information required to reanalyze the data reported in this paper is available from the lead contact upon request.
