# Supplementary material for: Loss of central mineralocorticoid or glucocorticoid receptors impacts auditory nerve processing in the cochlea
Source: iScience. 2022 Feb 26;25(3):103981. doi: 10.1016/j.isci.2022.103981 (PMC8914323; doi:10.1016/j.isci.2022.103981)
Supplement: Document S1. Figures S1–S3 [file mmc1.pdf]

iScience, Volume *25*

Supplemental information

Loss of central mineralocorticoid or glucocorticoid receptors impacts auditory nerve processing in the cochlea

Philine Marchetta, Philipp Eckert, Robert Lukowski, Peter Ruth, Wibke Singer, Lukas Rüttiger, and Marlies Knipper

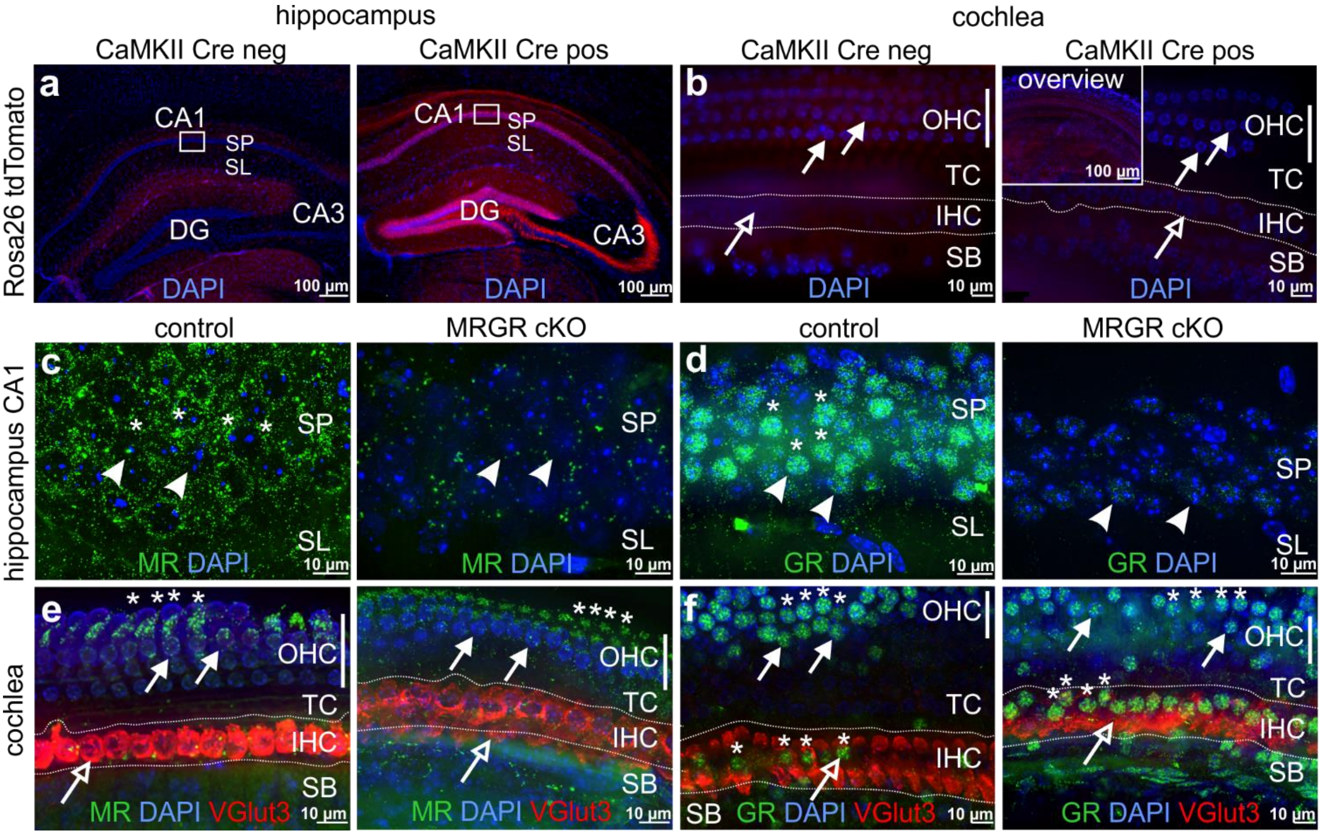

Supplement Figure 1: Expression of CaMKIIα, MR and GR in hippocampus and cochlea, Related to Figure 1.

(**a**) Expression of CaMKIIα (red) in the hippocampus of CaMKII-Rosa^{tdTomato} Cre positive mice, but not Cre negative mice. Scale bar = 100 µm.

(**b**) No expression of CaMKIIα in the Organ of Corti of CaMKII-Rosa^{tdTomato} Cre positive mice and negative mice. Open arrows = IHCs; closed arrows = OHCs.

(**c**) Expression of MR (green) in hippocampal pyramidal neurons in the stratum pyramidale (SP) of control mice, but not MRGR cKO mice. Arrow heads = pyramidal cells; * = MR expression (green).

(**d**) Expression of GR (green) in hippocampal pyramidal neurons in the SP of control mice, but not MRGR cKO mice. Arrow heads = pyramidal cells; * = GR expression (green).

(**e**) MR is expressed in cochleae of both MRGR cKO and control mice. Open arrows = IHCs; closed arrows = OHCs; * = MR expression (green).

(**f**) GR is expressed in cochleae of both MRGR cKO and control mice. Open arrows = IHCs; closed arrows = OHCs; * = GR expression (green).

SL = Stratum lucidum, DG = dentate gyrus, CA = cornu ammonis, OHC = outer hair cells, IHC = inner hair cells, TC = tunnel of Corti, SB = spiral bundle. DAPI nuclei staining = blue.

(**b**) – (**f**) Scale bar = 100 µm.

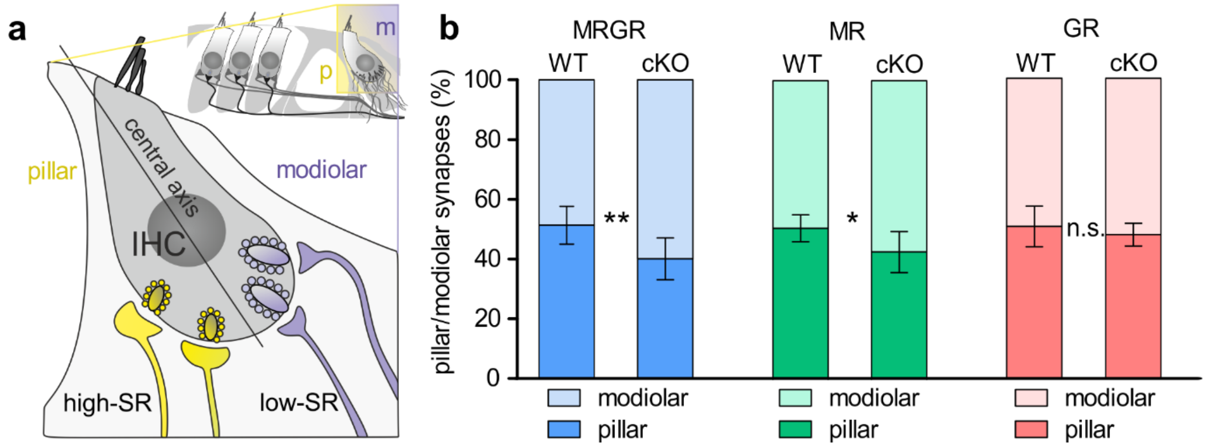

Supplementary Figure 2: Reduced numbers of pillar IHC ribbons in MRGR and MR cKO mice, Related to Figure 2.

(**a**) Schematic of the IHC-ANF synapses. Ribbon synapses that are located on the pillar side of the IHC (yellow) are small and have large postsynapses, that are associated with high-SR ANF (yellow). Modiolar ribbon synapses (violet) are relatively large and have contact to smaller postsynapses that belong to low-SR ANF (violet).

(**b**) In the midbasal cochlear turn, MRGR cKO mice like MR cKO mice had a lower percentage of pillar IHC ribbons compared with the corresponding control mice (unpaired Student's t-test, MRGR: t(15) = 3.57, $p = 0.0028$, WT: $n = 9$, KO: $n = 8$ mice; MR: t(10) = 2.38, $p = 0.0387$, $n = 6$ mice). GR cKO mice were not different in the pillar/modiolar gradient from control mice (unpaired Student's t-test, t(8) = 0.79, $p = 0.4551$, $n = 5$ mice).

Mean ± standard deviation (SD). * = $p < 0.05$, ** = $p < 0.01$, n.s. = not significant ($p > 0.05$).

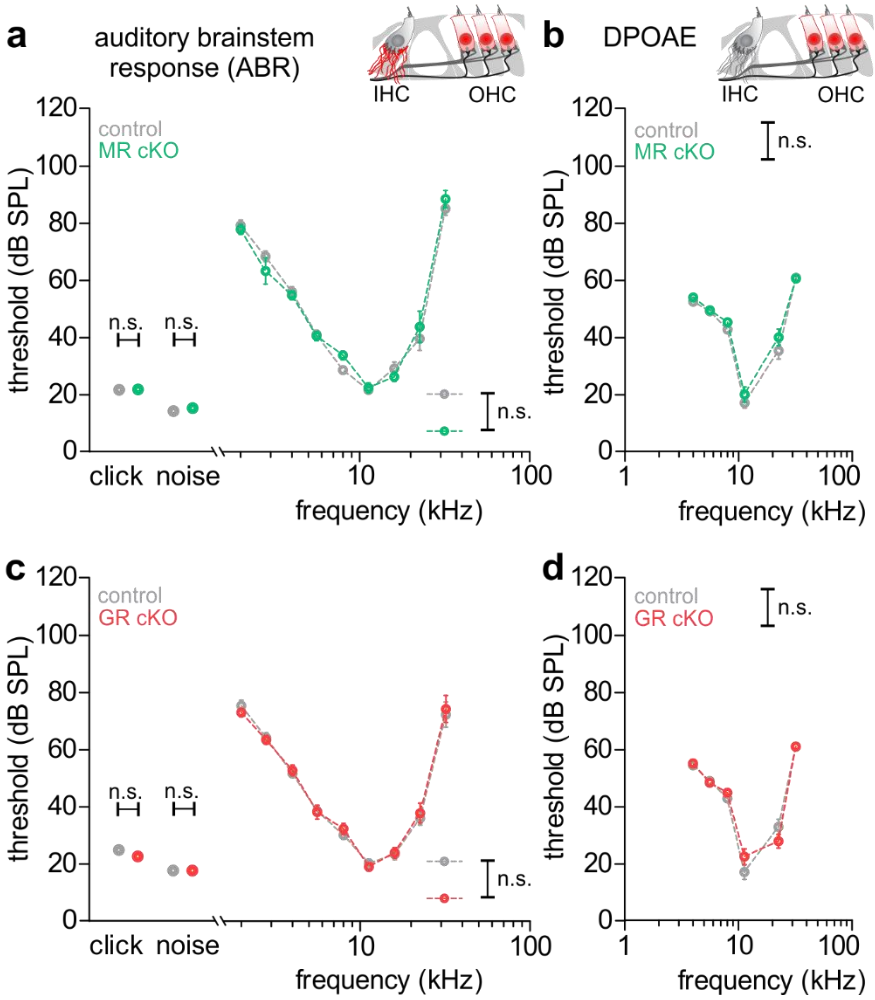

Supplementary Figure 3: No effect on outer hair cell function in MR cKO mice and GR cKO mice, Related to Figure 3 and 4.

(**a**) No difference of thresholds with click (unpaired Student's t-test, t(56)=0.07, p = 0.944, WT: n = 14/28, KO: n = 15/30 mice/ears) and noise-burst evoked ABR (unpaired Student's t-test, t(56) = 0.64, p = 0.519, WT: n = 14/28, KO: n = 15/30 mice/ears) as well as pure tone frequency ABR (2-way ANOVA, F(1,8) = 0.045, p = 0.832, WT: n = 14, KO: n = 15 mice) between controls mice and MR cKO mice.

(**b**) No difference of thresholds with DPOAE between MR cKO and control mice (2-way ANOVA, F(1,5) = 4.030, p = 0.455, WT: n = 14/28, KO: n = 15/30 mice/ears).

(**c**) No difference with click, and noise-burst, evoked and pure tone frequency ABR thresholds between control mice and GR cKO mice (click: t(58) = 1.398, p = 0.168, WT: n = 14/28, KO: n = 16/32 mice/ears, noise: t(58) = 0.124, p = 0.902, WT: n = 14/28, KO: n = 16/32 mice/ears, frequency: F(1,8) = 0.078, p = 0.781, WT: n = 13, KO: n = 16 mice).

(**d**) No difference at DPOAE thresholds between control mice and GR cKO mice (F(1,5) = 0.182, p = 0.670, WT: n = 14/28, KO: n = 16/32 mice/ears).

Mean ± standard error of the mean (SEM). n.s. = not significant (p > 0.05).
